# Self-Supervised Text-Vision Alignment for Automated Brain MRI Abnormality Detection: A Multicenter Study (ALIGN Study)

**DOI:** 10.1148/ryai.240619

**Published:** 2025-11-26

**Authors:** David A. Wood, Emily Guilhem, Sina Kafiabadi, Ayisha Al Busaidi, Kishan Dissanayake, Ahmed Hammam, Nina Mansoor, Matthew Townend, Siddharth Agarwal, Yiran Wei, Asif Mazumder, Gareth J. Barker, Peter Sasieni, Sébastien Ourselin, James H. Cole, Nikhil Nair, Anil Geetha, Chike Onyekwuluje, Rob Dineen, Permesh Dhillon, Carolyn Costigan, Kavi Fatania, Mark Igra, Rebecca Nichols, Janak Saada, Arne Juette, Ramona-Rita Barbara, Hilmar Spohr, Thomas C. Booth, for the MIDI Consortium Group

**Affiliations:** ^1^School of Biomedical Engineering and Imaging Sciences, King’s College London, Rayne Institute, 4th Floor, Lambeth Wing, London SE17 7EH, UK; ^2^King’s College Hospital NHS Foundation Trust, SE5 9RS, London, United Kingdom; ^3^Guy’s and St Thomas’ NHS Foundation Trust, SE1 9RT, London, United Kingdom; ^4^Department of Neuroimaging, Institute of Psychiatry, Psychology, & Neuroscience, King’s College London, SE5 9NU, United Kingdom; ^5^Wolfson Institute of Population Health, Queen Mary University of London, Charterhouse Square London, EC1M 6BQ, United Kingdom; ^6^Centre for Medical Image Computing, Department of Computer Science, University College London, W1W 7T, United Kingdom; ^7^Bedfordshire Hospitals NHS Foundation Trust, Bedford Hospital, South Wing, Kempston Road, Bedford, MK42 9DJ, United Kingdom; ^8^Radiological Sciences, School of Medicine, University of Nottingham, Nottingham, United Kingdom; ^9^University Hospitals NHS Trust, Nottingham, United Kingdom; ^10^Department of Neuroradiology, Floor B, Clarendon Wing, Leeds General Infirmary, Leeds, LS1 3EX, United Kingdom; ^11^Yeovil Hospital, Somerset NHS Foundation Trust, Yeovil, United Kingdom; ^12^Department of Radiology, Norfolk and Norwich University Hospital, Colney Lane, Norwich, Norfolk, NR4 7UY, United Kingdom; ^13^Members of the MIDI Consortium Group are listed at the end of the article.

**Keywords:** Head and Neck, Unsupervised Learning, Convolutional Neural Network (CNN), Neuroradiology

## Abstract

**Purpose:**

To develop a self-supervised text-vision framework to detect abnormalities on brain MRI scans by leveraging free-text neuroradiology reports, eliminating the need for expert-labeled training datasets.

**Materials and Methods:**

This retrospective and prospective multicenter study included 81 936 brain MRI examinations and corresponding radiology reports for adult patients at two UK National Health Service hospitals from January 2008 to December 2019 for training and internal testing and 1369 prospectively collected examinations between March 2022 and March 2024 from four separate National Health Service hospitals for external testing (ClinicalTrials.gov no. NCT04368481). A neuroradiology language model (NeuroBERT) was trained using self-supervised tasks to generate report embeddings. Convolutional neural networks (one per MRI sequence) were trained to map scans to embeddings by minimizing mean squared error loss. The framework then detected abnormalities in new examinations by scoring scans against query sentences using text-image similarity. Model diagnostic performance was assessed using the area under the receiver operating characteristic curve (AUC).

**Results:**

The framework achieved an AUC of 0.95 (95% CI: 0.94, 0.97) for normal versus abnormal classification and generalized to external sites with examination-level AUCs of 0.90 (95% CI: 0.86, 0.93) in Bedford, 0.87 (95% CI: 0.83, 0.90) in Nottingham, 0.86 (95% CI: 0.83, 0.90) in Norwich, and 0.85 (95% CI: 0.81, 0.89) in Yeovil. In five zero-shot classification tasks—acute stroke, multiple sclerosis, intracranial hemorrhage, meningioma, and hydrocephalus—the framework achieved a mean AUC of 0.89 (range, 0.77–0.93). For visual-semantic image retrieval, mean precision was 0.84 among the top 15 images across seven pathologies.

**Conclusion:**

The self-supervised text-vision framework accurately detected brain MRI abnormalities without expert-labeled datasets.

Clinical trial registration no. NCT04368481

**Keywords:** Head and Neck, Unsupervised Learning, Convolutional Neural Network (CNN), Neuroradiology

© The Author(s) 2025. Published by the Radiological Society of North America under a CC BY 4.0 license.

*Supplemental material is available for this article.*

See also commentary by Ghodasara in this issue.

## Graphical abstract

**Figure:**
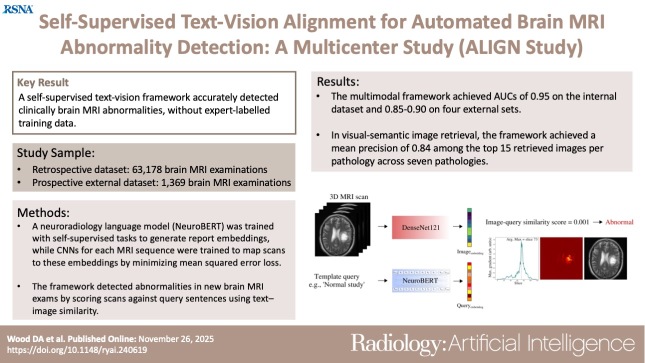

**Summary**

A self-supervised text-vision framework accurately detected clinically relevant brain MRI abnormalities without expert-labeled training data, potentially enabling automated triage, clinical decision support, and educational applications.

**Key Points**

■ A multimodal framework trained on 63 178 unlabeled brain MRI examinations achieved an area under the receiver operating characteristic curve (AUC) of 0.95 (95% CI: 0.94, 0.97) for classification of normal versus abnormal findings and generalized well to prospectively collected data from four external hospitals, with AUCs of 0.85–0.90.
■ In visual-semantic image retrieval, the framework achieved a mean precision of 0.84 among the top 15 retrieved images per pathology across seven pathologies, enabling retrieval based on textual descriptions.
■ By combining classification and retrieval, the framework potentially supports applications in automated triage, clinical decision support, and education.

## Introduction

MRI is essential for diagnosing and managing various neurologic conditions (1). However, increasing demand for brain MRI examinations and a global shortage of radiologists are straining health care systems. Radiologists often cannot complete reports within contracted hours, leading to substantial delays (2) and raising concerns about fatigue-related diagnostic errors (3). These issues result in treatment delays, poorer patient outcomes, and higher health care costs (4).

Artificial intelligence could alleviate pressure on radiology departments by supporting real-time triage of examinations (5–11) or assisting radiologists in reducing report errors. Traditional artificial intelligence models rely on deep learning trained on expert-labeled datasets (12–15), although this approach has limitations. First, it is increasingly unjustifiable to use radiologists’ time for manual image annotation, making large, representative training datasets a bottleneck (16–18). Second, supervised learning with categorically labeled data restricts classification to a fixed set of classes, requiring additional labeled examples whenever new tasks emerge—a substantial problem in neuroradiology, in which clinical demands constantly evolve (19).

Automated report-annotation methods that extract labels from free-text radiology reports have been proposed to address this bottleneck (20–24). However, these approaches still require considerable expert input to define categories and perform dataset annotation for report classifier training; yield inflexible, fixed label sets; and collapse rich descriptive language (including lesion location and severity) into coarse classes, discarding valuable supervisory detail.

These challenges have spurred interest in multimodal self-supervised methods that enable computer vision models to learn directly from free-text radiology reports (25,26). Reports contain detailed descriptions by expert radiologists and are stored alongside imaging data on hospital picture archiving and communication systems, making them readily available. However, applications of self-supervised methods have been largely limited to chest radiographs (27,28), partly due to open-access datasets such as MIMIC-CXR (29). To the best of our knowledge, no prior work demonstrates text-vision models for brain abnormality detection or for the complex modality of MRI (30).

In this study, we present a self-supervised text-vision framework that learns to detect clinically relevant abnormalities from unlabeled hospital brain MRI scans. Our two-step training involves training NeuroBERT, a dedicated neuroradiology language model, to generate embeddings of reports via domain-specific self-supervised learning tasks and training convolutional neural networks (CNNs)—one per MRI sequence type, covering all routine sequences—to map individual brain scans to their corresponding text vector representations by minimizing mean squared error loss. Once trained, the framework detected abnormalities in unreported brain MRI examinations by scoring scans against query sentences using text-image similarity.

## Materials and Methods

This retrospective and prospective study was approved by the UK National Health Research Authority (IRAS 235658) and research ethics committee (REC 18/YH/0458). The requirement for informed consent was waived for the retrospective data; participants in the prospective study provided written informed consent. The study was registered with ClinicalTrials.gov (no. NCT043681). No industry authors contributed. Two authors (D.A.W. and T.C.B.) controlled and analyzed data.

### Data

Inclusion criteria were patients aged 18 years or older with brain MRI scans. Exclusion criteria were poor image quality or absence of a corresponding radiologist report and, for the retrospective cohort, lack of consent for future research in the historic database.

To maximize clinical utility, we focused on the most commonly performed MRI sequences at UK National Health Service (NHS) hospitals: axial T2-weighted, axial diffusion-weighted imaging, coronal T2-fluid-attenuated inversion recovery, axial gradient-recalled echo T2*-weighted, coronal T1-weighted, axial T1-weighted postcontrast, axial T2 fluid-attenuated inversion recovery, and coronal T1-weighted postcontrast sequences (Fig S1, Appendix S1). Only examinations including at least one of these sequences were included for training and testing.

We obtained 81 936 consecutive brain MRI examinations for patients aged 18 years or older performed at King’s College Hospital NHS Trust and Guy’s and St Thomas’ NHS Trust between January 2008 and December 2019. These large NHS hospitals cover all neurologic conditions and serve a diverse patient population (up to 40% non-White). Examinations were performed on Signa 1.5-T HDX (GE HealthCare), Aera 1.5-T (Siemens), Avanto 1.5-T (Siemens), Ingenia 1.5-T (Philips Healthcare), Intera 1.5-T (Philips), or Skyra 3-T (Siemens) scanners. The corresponding neuroradiology reports, produced by expert neuroradiologists, were also obtained. Reports were largely unstructured, typically comprising five to 10 sentences of image interpretation, sometimes including clinical history comments and recommendations. All data were de-identified.

To assess generalizability of the framework to diverse MR image data characteristics from independent external sites, we prospectively and consecutively collected 1369 examinations between March 2022 and March 2024 from four geographically distinct external UK hospitals: Yeovil, Nottingham, Bedford, and Norwich. These external datasets captured real-world variability in imaging protocols, scanner field strengths, models, and manufacturers as well as differences in participant demographics. Detailed information on these datasets is provided in Appendix S2.

### Training and Testing Datasets

Before model training, several test sets were generated with reference-standard labels to evaluate classification performance. All labeling was performed by three expert neuroradiologists (E.G., with 5 years as a consultant neuroradiologist; and S.K. and A.A.B., with 7 years as consultant neuroradiologists [the UK consultant grade is equivalent to a US attending]) who assessed all available image sequences, with consensus decisions reached in cases of disagreement. First, a test set of 800 examinations was labeled as normal or abnormal using predefined criteria (Appendix S3). Briefly, findings that could generate a downstream clinical intervention were labeled as abnormal (referral for multidisciplinary team discussion was considered the minimal intervention); this included findings excessive for age (eg, excessive volume loss).

Next, five specialized test sets of 200 examinations each were created: acute stroke versus no acute stroke, multiple sclerosis versus no multiple sclerosis, intracranial hemorrhage versus no intracranial hemorrhage, meningioma versus no meningioma, and hydrocephalus versus no hydrocephalus. Each had 100 positive and 100 negative examinations. The negative category included normal examinations and a random selection of other abnormalities, reflecting real-world practice. These categories were chosen for their clinical significance and diversity.

**Figure 1:**
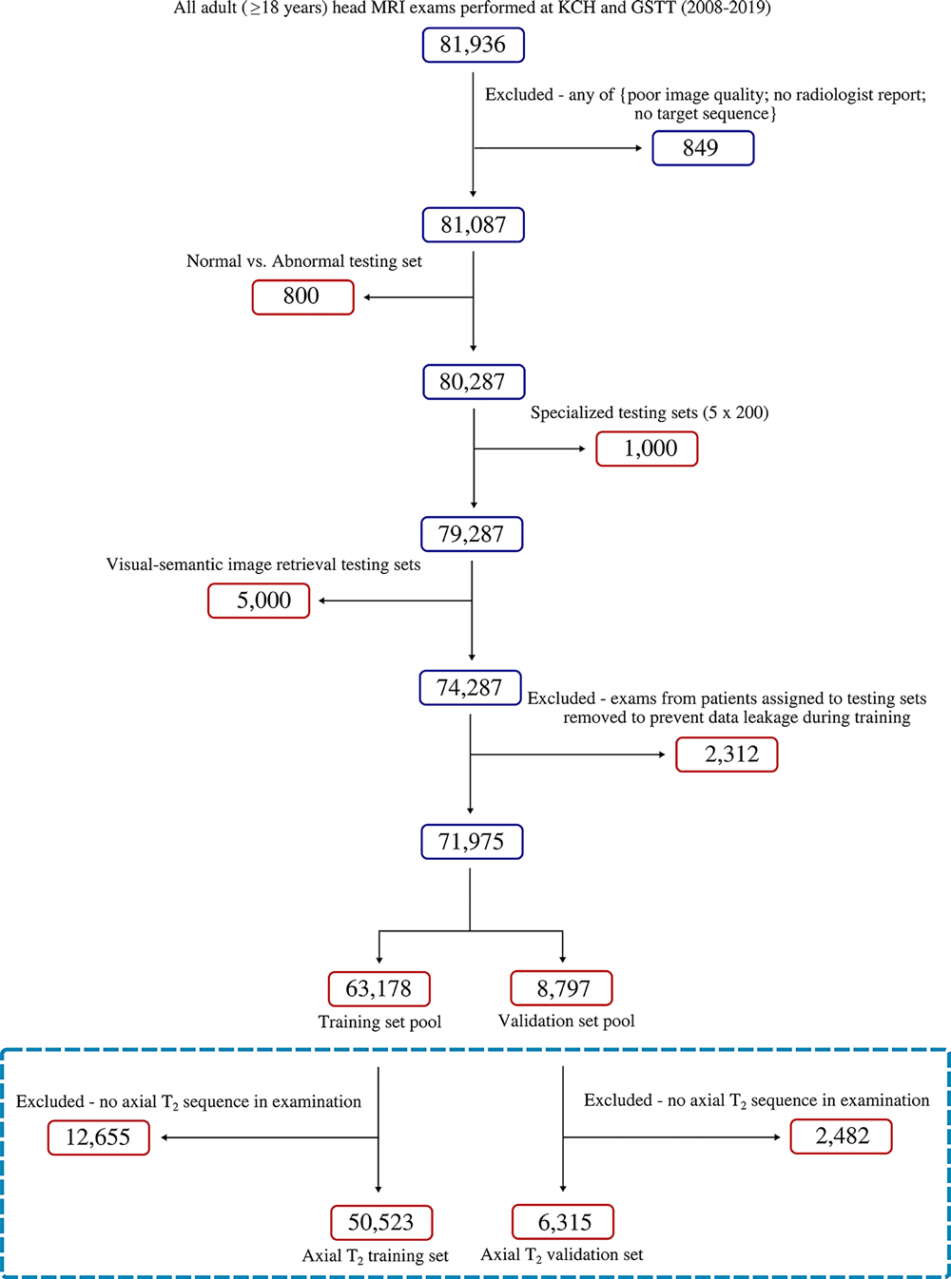
Flowchart shows datasets used to train, validate, and test the text-vision framework. Note that the training and validation set splitting procedure shown in the dashed blue box was performed separately for each of the eight MRI sequences included in this study; however, only a single sequence (axial T2) has been included here for brevity. GSTT = Guy’s and St Thomas’ NHS Trust, KCH = King’s College Hospital NHS Foundation Trust.

Finally, a holdout set of 5000 unlabeled examinations was set aside to evaluate visual-semantic image retrieval. All remaining examinations were split into unlabeled training (85%) and validation (15%) datasets for model development. Data were split at the participant level to avoid data leakage (ie, all examinations of the same participant, including follow-up imaging, were only present in a single dataset). Further dataset details are provided in Table 1 and Figure 1.

**Table 1:** Training, Validation, and Testing Datasets

MRI Sequence	No. in Training Dataset	No. in Validation Dataset	No. in Testing Dataset						
			Normal or Abnormal	Acute Stroke	Multiple Sclerosis	Hemorrhage	Meningioma	Hydrocephalus	Database Search
Axial T2	50 523	6315	800	200	200	200	200	200	5000
Axial DWI	42 220	5246	718	200	178	183	188	183	4398
Coronal T2-FLAIR	28 351	3518	451	121	118	132	138	122	2811
Axial GRE	20 613	2605	384	116	102	200	100	110	2102
Coronal T1	11 080	1365	169	78	81	73	71	92	1190
Axial T1 (postcontrast)	10 695	1329	151	74	112	70	78	61	1015
Axial T2-FLAIR	9582	1236	136	59	55	63	61	58	934
Coronal T1 (postcontrast)	7736	976	109	48	88	39	32	29	878

Note.—DWI = diffusion-weighted imaging, FLAIR = fluid-attenuated inversion recovery, GRE = gradient-recalled echo.

For external evaluation, normal versus abnormal labels for the prospectively collected examinations from four independent UK hospitals (Yeovil, Nottingham, Bedford, and Norwich) were provided by expert neuroradiologists, who applied the same rigorous method as the internal labeling process, interrogating all available sequences for each examination and reaching consensus in cases of disagreement.

### Brain MRI Preprocessing

Minimal automated preprocessing was performed. Three-dimensional MRI scans in Digital Imaging and Communications in Medicine format were converted to Neuroimaging Informatics Technology Initiative, resampled to a common voxel size (1 mm^3^), and cropped or padded to a common array size (180 × 180 × 180 mm). Intensities were normalized by subtracting the mean and dividing by the SD. To maintain real-time clinical utility, computationally expensive steps, such as bias-field correction and spatial registration, were avoided. Skull stripping was not performed to allow detection of extracranial abnormalities. Digital Imaging and Communications in Medicine files were loaded using Pydicom and converted to Neuroimaging Informatics Technology Initiative format with dcm2niix (31). Project MONAI (32) was used to resample, resize, and normalize each image.

### Self-Supervised Text-Vision Framework

Our self-supervised text-vision framework consisted of a dedicated neuroradiology language model (NeuroBERT) and CNN-based image encoders.

**NeuroBERT model development**

**Figure 2:**
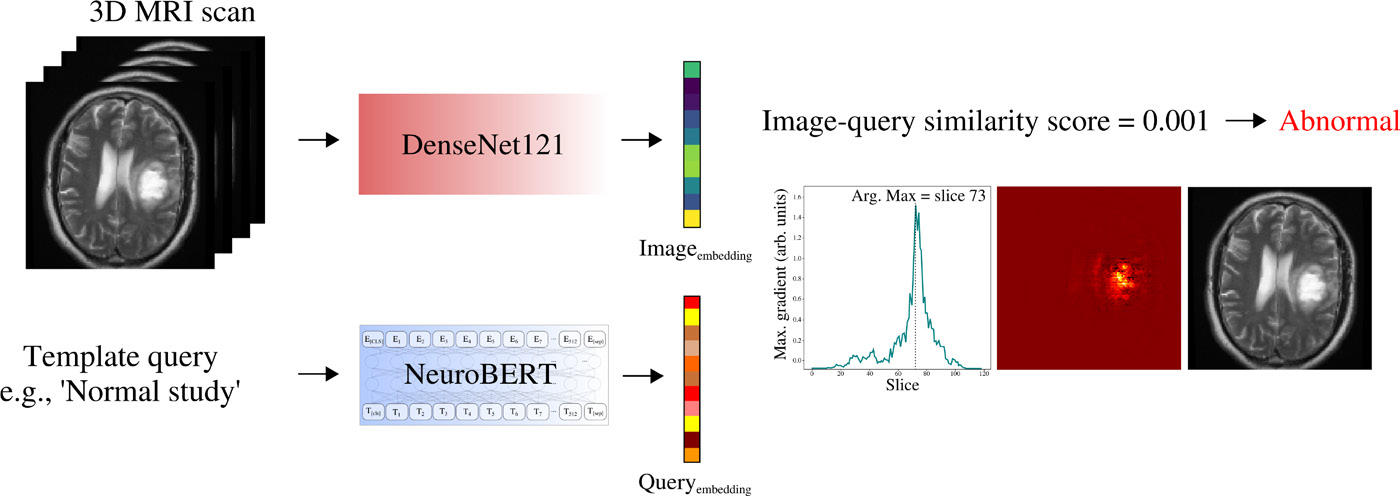
Overview of the text-vision brain abnormality detection framework. A convolutional neural network encoder maps three-dimensional (3D) brain MRI scans to image embeddings in a semantic space learned from neuroradiology reports via a dedicated language model (NeuroBERT). Abnormality detection is achieved by scoring scans against query sentences using text-image similarity. In the example, a scan with an extra-axial mass scored against ‘normal study’ yields a low similarity score, indicating abnormality; the saliency map highlights its location. The framework can generate similarity scores for arbitrary findings, helping identify the nature of abnormalities. Arb = arbitrary, arg = argument, max = maximum.

NeuroBERT serves two main functions: It generates embeddings of neuroradiology reports for training our computer vision models, and at test time, it embeds query sentences to detect abnormalities in unseen scans (Fig 2). Architecturally identical to BERT (33), a state-of-the-art transformer language model, NeuroBERT required key modifications to optimize it for neuroradiology reports.

**Custom neuroradiologic vocabulary**

Transformer-based language models rely on tokenization, splitting text into units and converting them to machine-readable IDs. BERT uses WordPiece tokenization (34), assigning unique IDs to common words but breaking rarer words into subword pieces. However, this poses a problem with complex neuroradiologic text, in which many commonly used terms are broken into irrelevant pieces (eg, *hemorrhage* becomes *he, morr, hage*). To address this, a custom WordPiece vocabulary of 10 000 words was trained using our corpus of unlabeled neuroradiology reports. Although the original BERT vocabulary could be extended by adding domain-specific tokens (eg, assigning neuroradiologic terms such as *hemorrhage* to indexes beyond the original vocabulary), this approach produces a hybrid tokenization scheme in which some specialized words are split while others remain whole. By training from scratch, we ensured that our custom vocabulary fully reflects the language of neuroradiology and reduces unnecessary word fragmentation compared with general-purpose vocabularies (Appendix S4).

**Masked language modeling without next sentence prediction**

BERT was trained with two self-supervised tasks: next sentence prediction and masked language modeling. However, in neuroradiology reports, adjacent but unrelated sentences make next sentence prediction inappropriate. We therefore omitted this task and initially trained NeuroBERT with masked language modeling only (Fig S2, Appendix S4). We randomly replaced 15% of tokens with the <mask> token. Short sequences were input into NeuroBERT, which generated 768-dimensional contextualized word embeddings via stacked self-attention layers. The <mask> embeddings were passed through a feedforward network with a softmax layer to produce a probability distribution over the custom vocabulary. Cross-entropy loss between predictions and ground truth tokens was computed, and model weights were updated by gradient backpropagation.

**Radiology report section matching**

While masked language modeling generates rich contextualized word embeddings, we require fixed-dimensional vector representations of entire reports. Inspired by prior work (26), NeuroBERT was trained to perform radiology section matching, encouraging similar embeddings for Findings and Summary sections from the same report. Findings sections describe observed abnormalities and those ruled out, whereas Summary sections provide concise recapitulations for clinicians. To match these sections, NeuroBERT had to identify and prioritize relevant information in embeddings. Our analysis in Appendix S4 confirmed that this unified embedding reliably captured all key abnormalities, even when a report includes multiple distinct findings.

Using the twin network architecture of SentenceBERT (35), two copies of NeuroBERT with tied weights were created. Findings and Summary sections from true pairs (same report) or false pairs (random reports) were tokenized and input into the network. Mean pooling generated section-level embeddings, and a cosine similarity score was calculated. Mean squared error was used between the similarity score and ground truth label (1 for true pairs, 0 for false pairs) as the training objective (Fig S3, Appendix S4). After radiology section matching training, NeuroBERT generated embedding-based summaries from reports, serving as latent, high-dimensional representations for computer vision training. The complete report was used to generate these summaries, ensuring that all clinically relevant information was captured regardless of report formatting. Trained NeuroBERT model, custom vocabulary, and tokenizer are publicly available at *https://huggingface.co/davvwood/NeuroBERT*.

**CNN image encoder**

The CNN image encoders mapped three-dimensional brain MRI scans to embeddings in the same semantic space as NeuroBERT. Because MRI examinations comprise multiple sequences, separate CNN models were developed for the commonly performed sequences. Each single-sequence model is based on a modified DenseNet121 architecture (36). The four dense blocks and transition layers were retained, although the final linear layer and softmax were removed. Global average pooling was applied to the output, which was concatenated with the participant’s age—critical for defining abnormality in neuroimaging because many findings (eg, white matter hyperintensities or atrophy) are abnormal only if they exceed what is expected for a given chronological age—and fed into a single-layer feedforward neural network to generate 768-dimensional age-conditional image embeddings. Without age information—which is always included in MRI data—an older participant could be incorrectly classified as abnormal because of normal, age-appropriate volume changes. Although additional metadata (eg, sex, scanner parameters, and other clinical variables) could serve as covariates, they are often unavailable or incomplete and do not directly determine abnormality.

**Figure 3:**
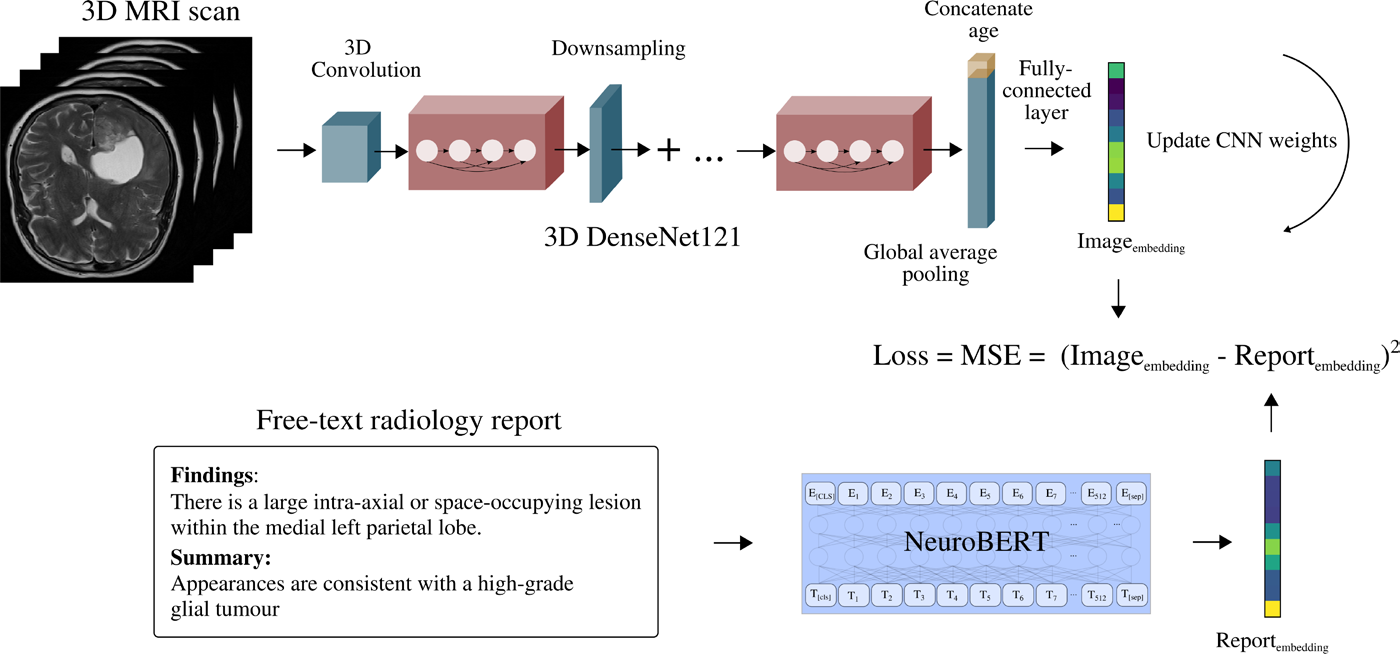
Overview of the computer vision training procedure. Single-sequence image encoders (based on DenseNet121) map three-dimensional (3D) brain MRI scans to embeddings in the same semantic space as NeuroBERT. The encoders are trained by minimizing the mean squared error (MSE) loss between image embeddings and report embeddings generated by NeuroBERT from corresponding reports. CNN = convolutional neural network.

During training, batches of paired MRI scans and corresponding reports were input into the image encoder and NeuroBERT, respectively (Fig 3). The training objective was elementwise mean squared error loss between image and report embeddings; backpropagation updated the image encoder weights, with NeuroBERT’s parameters kept constant.

This two-step training procedure can be considered cross-modal distillation, in which a pretrained model in one modality (NeuroBERT) served as the teacher, and its frozen embeddings supervised a student network in another modality (the sequence-specific CNNs).

**Brain abnormality detection in unreported scans**

At test time, abnormalities in unreported MRI examinations were detected by treating the problem as a text-image similarity task, scoring scans against query sentences. Test images were input into the image encoder to generate image embeddings. Expert-derived query sentences were processed by NeuroBERT to generate text embeddings. Cosine similarity—the normalized dot product of image and text embeddings—was calculated, yielding a similarity score between 0 and 1 (negative values were set to 0) that captured semantic alignment (higher scores indicated greater alignment; Appendix S5). These scores served as pseudoprobabilities for classification decisions and for generation of receiver operating characteristic curves.

Detailed training and model interpretability information is provided in Appendix S6.

### Classification Performance Evaluation

Classification performance was evaluated in two ways: normal versus abnormal classification for triage applications and five specialized diagnostic tasks: acute stroke versus no acute stroke, multiple sclerosis versus no multiple sclerosis, intracranial hemorrhage versus no intracranial hemorrhage, meningioma versus no meningioma, and hydrocephalus versus no hydrocephalus. This was zero-shot classification, as the model was not explicitly trained on these categories but leveraged contextual understanding from self-supervised learning.

For normal versus abnormal classification, all sequences for each examination were scored against the query sentence “There are normal intracranial appearances.” The resulting normality scores were subtracted from 1 to obtain abnormality scores in the range [0,1]. Performance was reported for each sequence, and an ensemble strategy that returned the highest single-sequence abnormality score per examination was investigated, analogous to clinical practice.

To assess generalizability, the same testing procedure was applied to prospectively collected data from the four external UK hospitals (Yeovil, Nottingham, Bedford, and Norwich). For each external examination, all available sequences were scored using the same approach, and the ensemble strategy was applied to generate examination-level predictions. Subgroup analyses evaluated performance across sex, age, MRI field strength, scanner manufacturer, and scanner model at each site. Detailed information regarding these external datasets and subgroup distributions are provided in Appendix S2.

For specialized tasks, sequences were scored against expert-derived query sentences: “There is restricted diffusion in keeping with acute infarction,” “Appearances meet the McDonald criteria for multiple sclerosis,” “Appearances are in keeping with a recent bleed,” “Appearances are in keeping with a meningioma,” and “Appearances are in keeping with hydrocephalus,” respectively. The ensemble approach was used to generate examination-level predictions. Appendix S4 presents a detailed analysis of NeuroBERT’s robustness to variations in prompt phrasing.

### Visual-Semantic Image Retrieval

Visual-semantic image retrieval involves retrieving images from a dataset that align with textual descriptors. Performance was evaluated using the holdout test set of 5000 examinations. Three expert neuroradiologists (E.G., S.K., and A.A.B.) selected seven relevant pathologies (glioma, Alzheimer disease, pineal cyst, metastases, postsurgical resection cavity, hematoma, vestibular schwannoma) and formulated corresponding text queries. Each query was encoded with NeuroBERT, and images were encoded with the relevant CNN encoders. Candidates were ranked by cosine similarity to the query, with higher scores indicating greater semantic alignment. For each query, the top 15 images were retrieved, and retrieval precision was measured: the fraction of those images that were truly relevant. This represented a special case of the widely used precision@K metric, here with *K* = 15. When *K* is small (eg, 1–3), precision can appear artificially high because only the most confident examples are considered; selecting *K* = 15 balanced avoidance of such inflation with maintenance of a manageable review set size under clinical time constraints (Appendix S5).

### Statistical Analysis

Performance was quantified using the area under the receiver operating characteristic curve (AUC), with 95% CIs computed with the Wald normal-approximation (CI = AUC ± 1.96 √ [AUC × (1 – AUC) / N]), along with macroaveraged precision, recall, and F1 score. The DeLong test (37) was used to assess statistical significance between single-sequence and ensemble approaches. *P* value less than .05 indicated a statistically significant difference. Saliency lineouts and heat maps were generated with guided backpropagation to visualize influential image regions. All analyses were conducted using Python, version 3.11 (Python Software Foundation).

## Results

### Dataset Characteristics

The internal test dataset for the normal versus abnormal task included 800 patients (mean age, 50.7 years ± 17.4 [SD]; 427 female). The five specialized internal test datasets each included 200 patients (mean age, 54.2 years ± 18.5; 522 female). The external prospective dataset included 1369 participants (mean age, 57.1 years ± 18.4; 797 female).

### Normal versus Abnormal Classification

**Figure 4:**
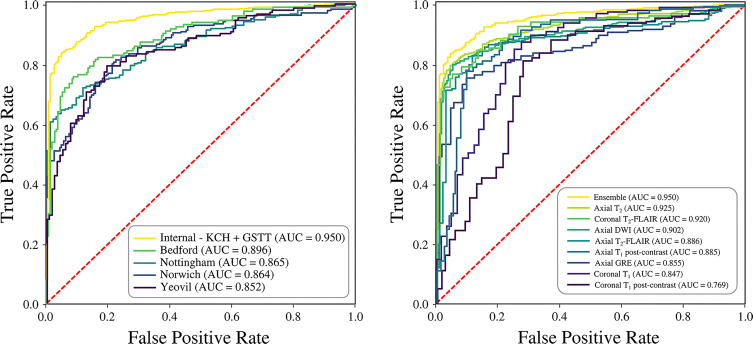
Left: Receiver operating characteristic (ROC) curves for our eight single-sequence text-vision models on the normal versus abnormal classification task, along with an ensemble approach which returns the highest single-sequence abnormality score for each examination. In order of descending performance, the areas under the ROC curves (AUCs) were: ensemble 0.95 (95% CI: 0.94, 0.97), axial T2-weighted 0.93 (95% CI: 0.91, 0.94), coronal T2-FLAIR 0.92 (95% CI: 0.90, 0.95), axial DWI 0.90 (95% CI: 0.88, 0.92), axial T2-FLAIR 0.89 (95% CI: 0.83, 0.94), axial T1 (postcontrast) 0.89 (95% CI: 0.83, 0.94), axial GRE 0.86 (95% CI: 0.82, 0.89), coronal T1 0.85 (95% CI: 0.79, 0.90), and coronal T1 (postcontrast) 0.77 (95% CI: 0.69, 0.85). Right: When evaluated on four external UK hospitals, the ensemble approach achieved AUCs of 0.90 (95% CI: 0.86, 0.93) at Bedford, 0.87 (95% CI: 0.83, 0.90) at Nottingham, 0.86 (95% CI: 0.83, 0.90) at Norwich, and 0.85 (95% CI: 0.81, 0.89) at Yeovil, demonstrating robust generalizability despite heterogeneity in imaging protocols and patient populations. GSTT = Guy’s and St Thomas’ NHS Trust, KCH = King’s College Hospital NHS Foundation Trust.

**Figure 5:**
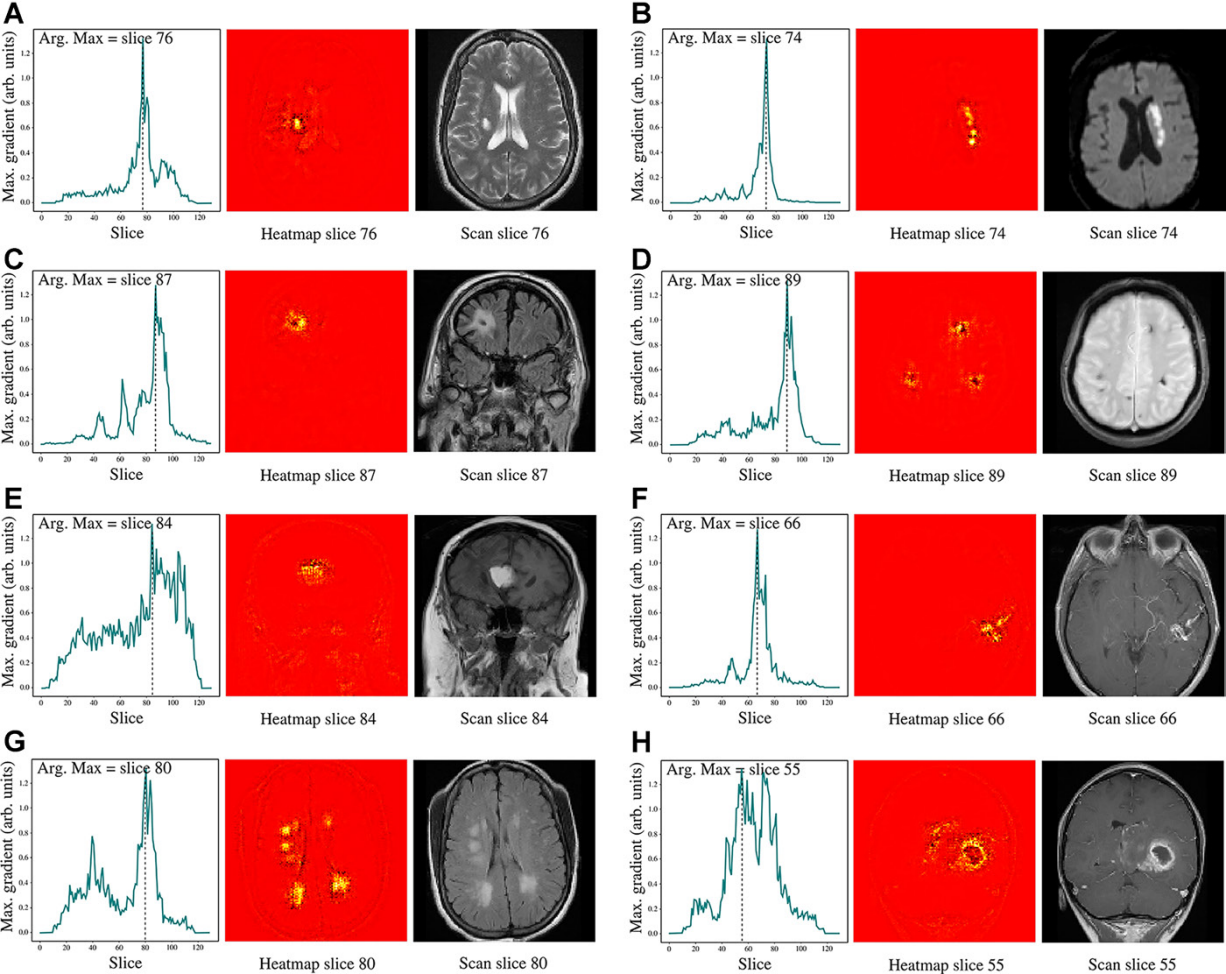
Smooth guided backpropagation visualizes regions most influential for our model’s predictions, accurately localizing various abnormalities across single-sequence models. Clockwise from top left: **(A)** chronic infarct on axial T2-weighted imaging; **(B)** acute infarction on axial DWI; **(C)** encephalomalacia on coronal T2-FLAIR; **(D)** microhemorrhages on axial GRE; **(E)** lipoma on coronal T1-weighted imaging;** (F)** arteriovenous malformation on axial T1-weighted postcontrast imaging; **(G)** severe small vessel disease on axial T2-FLAIR; **(H)** high-grade glioma on coronal T1-weighted postcontrast imaging. Left panels show saliency across sections; middle panels show heatmaps of the most influential section; right panels show the corresponding scan section. Note: Radiologic left and right are used. Arb = arbitrary, arg = argument, max = maximum.

**Figure 6:**
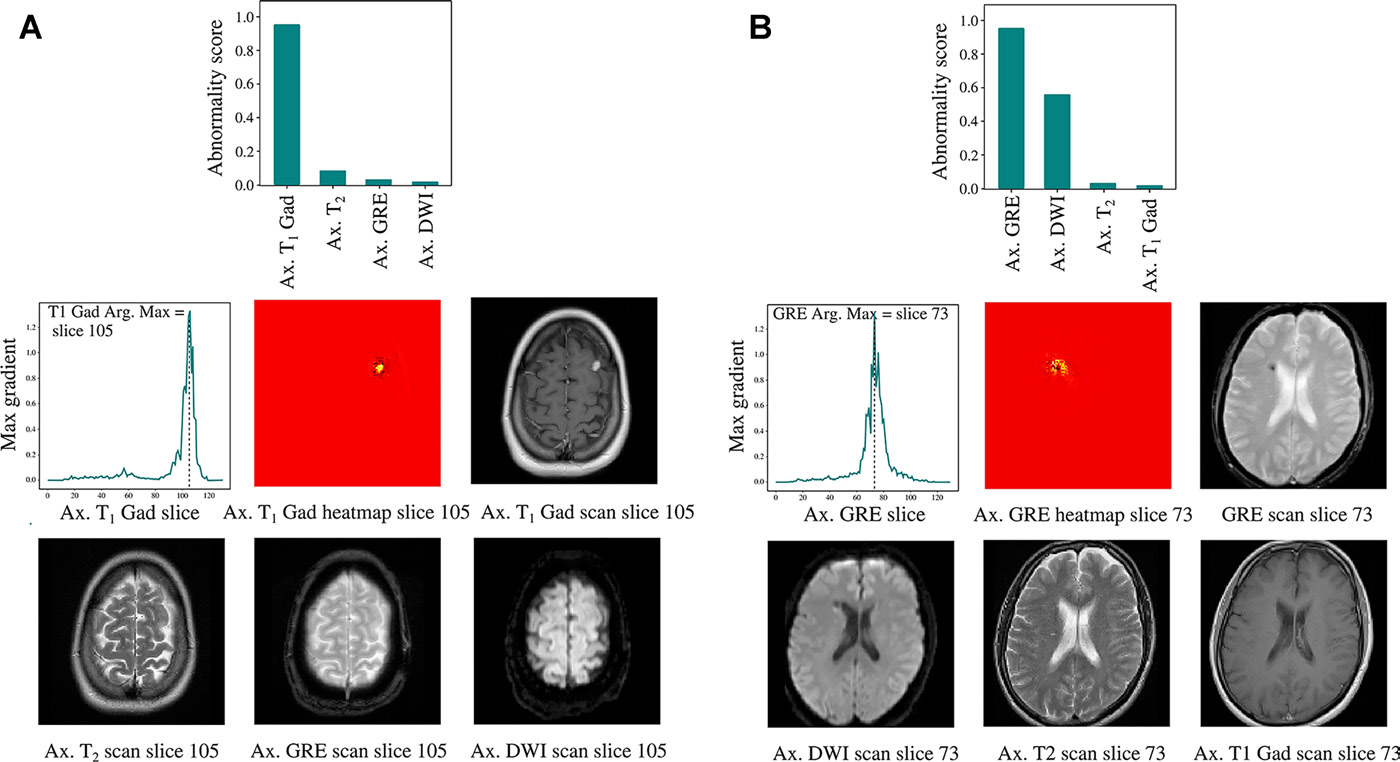
Visualization of our ensemble approach to brain abnormality detection. Abnormality scores are calculated for all available MRI sequences using the relevant single-sequence model. These scores are then ranked from high to low and displayed as a bar chart (top row), with the highest score taken as the examination-level prediction. Saliency lineouts and heatmaps highlighting the most influential image regions for the most important sequence are also generated (middle row). Our ensemble approach ensures that examinations containing abnormalities which are conspicuous on certain sequences and less visible on others are correctly classified. For example, the examination in panel **A** contains a left-sided metastasis which is visible only on the contrast-enhanced T1-weighted sequence. Likewise, the examination in panel **B** contains a small right-sided microhemorrhage which is visible only on the GRE and DWI sequences. This differential visibility is reflected in the sequence abnormality scores. Note: Using radiologic left and right. Arg = argument, Ax = axial, max = maximum.

Accurate classification of examinations as normal or abnormal was achieved for all single-sequence models (Table 2, Fig 4), with the best performance observed for axial T2-weighted sequences (AUC, 0.93; 95% CI: 0.91, 0.94). Examples of correctly identified abnormalities, with saliency lineouts and heat maps, are provided in Figure 5. Classification performance improved (*P* < .001) when using an ensemble strategy that returned the highest abnormality score across all sequences (AUC, 0.95; 95% CI: 0.94, 0.97), ensuring that examinations with abnormalities most conspicuous on certain sequences were classified correctly (Fig 6).

**Table 2:** Zero-Shot Classification Performance of Single-Sequence Vision Models on Normal versus Abnormal Binary Classification Task

MRI Sequence	AUC	Precision	Recall	F1 Score
Axial T2	0.93 (0.91, 0.94)	0.88	0.86	0.87
Coronal T2-FLAIR	0.92 (0.90, 0.95)	0.84	0.86	0.85
Axial DWI	0.90 (0.88, 0.92)	0.83	0.87	0.84
Axial T2-FLAIR	0.89 (0.83, 0.94)	0.80	0.85	0.82
Axial T1 (postcontrast)	0.89 (0.83, 0.94)	0.78	0.80	0.79
Axial GRE	0.86 (0.82, 0.89)	0.83	0.83	0.82
Coronal T1	0.85 (0.79, 0.90)	0.73	0.82	0.76
Coronal T1 (postcontrast)	0.77 (0.69, 0.85)	0.67	0.76	0.70
Ensemble	0.95 (0.94, 0.97)	0.88	0.88	0.88

Note.—Data in parentheses are 95% CIs. The zero-shot classification performance included the area under the receiver operating characteristic curve (AUC), macroaverage precision, macroaverage recall, and macroaverage F1 score. Classification performance improved (*P* < .001) when using an ensemble strategy that returned the highest abnormality score across all sequences. Importantly, the self-supervised text-vision approach achieved performance comparable to a conventional supervised DenseNet-121 (6) evaluated on the same *n* = 800 reference-standard test set (text-vision AUC, 0.95 vs supervised AUC, 0.94) while eliminating the need for costly manual annotation. DWI = diffusion-weighted imaging, FLAIR = fluid-attenuated inversion recovery, GRE = gradient-recalled echo.

When applied to external data from the four independent hospitals, the ensemble model achieved examination‐level AUCs of 0.90 (95% CI: 0.86, 0.93) in Bedford, 0.87 (95% CI: 0.83, 0.90) in Nottingham, 0.86 (95% CI: 0.83, 0.90) in Norwich, and 0.85 (95% CI: 0.81, 0.89) in Yeovil, demonstrating robust generalizability despite heterogeneity in imaging protocols, scanner types, and participant demographics (Fig 4). Subgroup analyses by sex, age, field strength, manufacturer, and scanner model are presented in Figures S5–S9.

### Specialized Abnormality Detection

Our framework accurately classified examinations in all five specialized categories (mean AUC across findings, 0.89; Table 3, Fig S10, Appendix S7). Examples of correctly classified examinations, with corresponding saliency visualizations, are shown in Figure S11.

**Table 3:** Zero-Shot Classification Performance for Five Specialized Abnormality Classification Tasks

Zero-Shot Classification Task	AUC	Precision	Recall	F1 Score
Acute stroke vs no acute stroke	0.94 (0.91, 0.98)	0.94	0.94	0.94
Multiple sclerosis vs no multiple sclerosis	0.94 (0.90, 0.97)	0.92	0.90	0.91
Intracranial hemorrhage vs no intracranial hemorrhage	0.89 (0.85, 0.93)	0.85	0.84	0.84
Meningioma vs no meningioma	0.88 (0.84, 0.93)	0.84	0.84	0.84
Hydrocephalus vs no hydrocephalus	0.81 (0.76, 0.86)	0.78	0.79	0.78

Note.—Data in parentheses are 95% CIs. The zero-shot classification performance included the area under the receiver operating characteristic curve (AUC), macroaverage precision, macroaverage recall, and macroaverage F1 score.

### Visual-Semantic Image Retrieval Performance

Accurate image retrieval was achieved for all seven pathologies (precision of retrieval when returning the top 15 results = 0.84) (Table 4). Examples of correctly retrieved images for each category are shown in Figure S12 in Appendix S8, along with their neuroradiology reports (not used for retrieval). In cases in which incorrect images were retrieved, the retrieved examinations often contained morphologically similar or ambiguous pathologies (Fig S13). Retrievals therefore supported radiologists wanting to see same or similar cases.

**Table 4:** Visual-Semantic Image Retrieval Performance

Abnormality	Precision of Retrieval When Returning Top 15 Results
Postsurgical resection cavity	0.93
Hematoma	0.92
Pineal cyst	0.87
Glioma	0.85
Vestibular schwannoma	0.83
Metastasis	0.76
Alzheimer disease	0.72

Note.—The precision of retrieval when returning the top 15 results is the fraction of those 15 images that truly contain the pathology of interest.

## Discussion

Demand for brain MRI and a global shortage of radiologists highlight the need for automated triage and decision-support systems. This study developed a self-supervised text-vision framework that learns from unlabeled brain MRI scans and their reports. In a triage task, the framework achieved an AUC of 0.95 for normal versus abnormal classification on the internal test set and generalized to four prospectively collected external sites with site AUCs of 0.90, 0.87, 0.86, and 0.85 in Bedford, Nottingham, Norwich, and Yeovil, respectively. The ensemble strategy outperformed the best single-sequence model (*P* < .001), supporting its use for examination-level prediction. In five specialized classification tasks—acute stroke, multiple sclerosis, intracranial hemorrhage, meningioma, and hydrocephalus—the mean AUC was 0.89. For visual-semantic image retrieval, the precision of retrieval when returning the top 15 results was 0.84 across seven pathologies.

Our work advances the field for brain MRI by learning from routine neuroradiology reports across common sequences and enabling zero-shot text-image similarity scoring of diverse findings and visual-semantic retrieval—all without manual image labels. Prior approaches have relied on supervised learning with categorically labeled images, obtained either manually or via automated report annotation—both require considerable expert involvement; impose rigid predefined classes that necessitate relabeling as new tasks emerge; and discard informative detail on appearance, location, and severity (12–24). In contrast, our self-supervised text-vision framework learns directly from free-text neuroradiology reports, aligning images and language in a shared embedding space to support zero-shot queries without predefined classes, preserve clinically rich semantics, and enable both classification and visual-semantic retrieval across routine sequences.

This study had limitations. First, the ensemble approach treated MRI sequences independently, which may be inadequate when diagnosis depends on combined findings across sequences. For example, differentiating a brain abscess from a high-grade tumor may require combining T1-weighted postcontrast enhancement with restricted diffusion on diffusion-weighted imaging. Second, single-sequence models mapped scans to examination-level embeddings, which could be problematic when findings were conspicuous on one sequence but subtle or invisible on another.

In conclusion, this study presented a self-supervised text-vision framework that detected clinically relevant abnormalities from unlabeled brain MRI scans, eliminating the need for expert-labeled datasets. Accurate zero-shot classification was demonstrated in both general and specialized categories, with potential applications in automated triage, clinical decision support, and educational training. A promising future application is detecting discrepancies in provisional radiology reports (Fig S14, Appendix S9): by comparing image embeddings with those of provisional reports, the system could flag inconsistencies or omissions for targeted review, which will require prospective clinical validation. Future work will also focus on developing joint multisequence modeling that explicitly captures intersequence dependencies.

## Supplemental Files

Appendices S1-S9, Figures S1-S14

Conflicts of Interest
