# Supplementary material for: Self-Supervised Text-Vision Alignment for Automated Brain MRI Abnormality Detection: A Multicenter Study (ALIGN Study)
Source: Radiol Artif Intell. 2025 Nov 26;8(2):e240619. doi: 10.1148/ryai.240619 (PMC13019336; doi:10.1148/ryai.240619)
Supplement: Appendices S1-S9, Figures S1-S14 [file ryai240619suppa1.pdf]

Supplemental materials undergo peer review but are not copyedited, and may include typographical errors.

©RSNA, 2026
10.1148/ryai.240619

Appendix S1

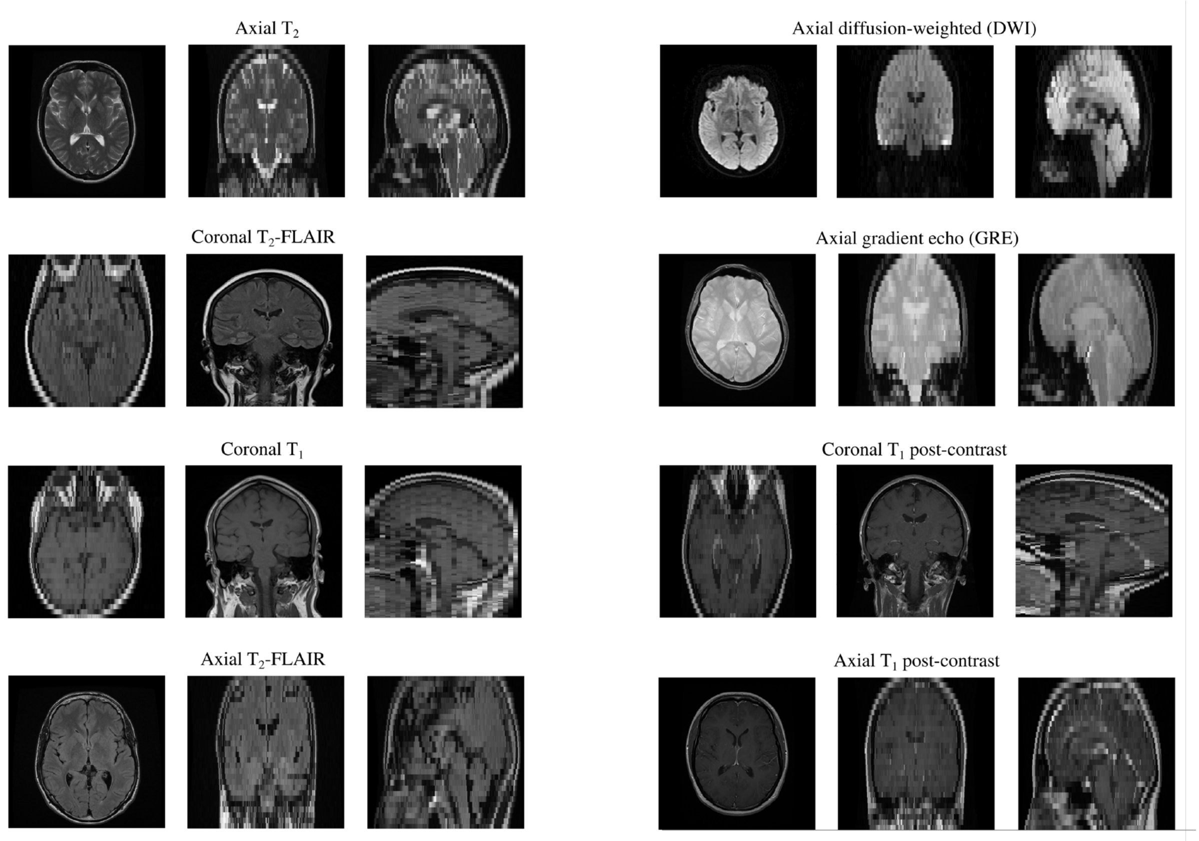

Figure S1: *Overview of the brain MRI scans used in this study. Clockwise from top left: axial T_2-weighted, axial diffusion-weighted (DWI), axial gradient echo (GRE), coronal T_1-weighted (post-contrast), axial T_1-weighted (post-contrast), axial T_2-FLAIR, coronal T_1-weighted, and coronal T_2-FLAIR scans. ADC maps were not consistently acquired in our dataset and are therefore not included. These sequences capture the majority of clinically relevant abnormalities. Although ADC maps could potentially enhance specificity for acute stroke detection, they were not included due to challenges associated with joint modelling—specifically, the need for precise spatial registration (particularly in abnormal anatomy), increased input dimensionality, and the consequent risk of overfitting.*

Supplemental materials undergo peer review but are not copyedited, and may include typographical errors.

Appendix S2

External testing datasets information

Overall examination numbers and age statistics:

Hospital	N	Age (mean ± SD)
Yeovil	294	63.0 ± 17.1
Nottingham	361	53.5 ± 16.7
Bedford	353	60.5 ± 17.9
Norwich	361	52.5 ± 19.5

Normal/Abnormal label distribution:

Hospital	Normal	Abnormal
Yeovil	131	163
Nottingham	92	269
Bedford	222	131
Norwich	178	183

Magnetic field strength distribution:

Hospital	1.5T	3T
Yeovil	294	-
Nottingham	269	92
Bedford	353	-
Norwich	361	-

Sex distribution:

Hospital	F	M
Yeovil	144	150
Nottingham	220	141
Bedford	210	143
Norwich	223	138

Supplemental materials undergo peer review but are not copyedited, and may include typographical errors.

Age distribution:

Hospital	< 40 years	40-60 years	> 60 years
Yeovil	47	79	168
Nottingham	84	138	139
Bedford	55	94	204
Norwich	115	98	148

MRI scanner manufacturer distribution:

Hospital	GE	Phillips	Siemens
Yeovil	-	294	-
Nottingham	18	84	259
Bedford	48	-	305
Norwich	117	-	244

MRI sequence distribution:

Hospital	Axial T2	Axial DWI	Axial FLAIR	Axial GRE	Coronal T1	Axial T1 (post contrast)	Coronal FLAIR	Coronal T1 (post contrast)
Yeovil	170	-	241	-	134	-	-	-
Nottingham	357	220	123	-	72	82	-	-
Bedford	330	226	310	-	2	34	-	-
Norwich	315	301	-	94	269	19	-	-

MRI scanner model distribution:

Yeovil:

Scanner Model	Count
Ingenia Ambition X	180
Ingenia	114

Supplemental materials undergo peer review but are not copyedited, and may include typographical errors.

Nottingham:

Scanner Model	Count
Aera	165
MAGNETOM Aera	94
Ingenia Elition X	74
SIGNA Premier	18
Ingenia	10

Bedford:

Scanner Model	Count
MAGNETOM Sola	157
Aera	148
SIGNA Voyager	48

Norwich:

Scanner Model	Count
Aera	208
SIGNA Voyager	117
MAGNETOM Sola	36

Supplemental materials undergo peer review but are not copyedited, and may include typographical errors.

Appendix S3

Brain abnormality definitions derived by a team of expert neuroradiologists (UK consultant grade; US attending equivalent) during consensus meetings which took place over the course of six months. A more detailed version is available in (6).

Binary abnormality definitions

Abnormal is defined as one or more abnormality described below.

Normal is defined as no abnormality described below.

Granular (specialized) abnormality definitions:

Small vessel disease

Fazekas and colleagues (38) gave a classification system for white matter lesions (WMLs) summarized as:

1. Mild - punctate WMLS: Fazekas I

2. Moderate - confluent WMLs: Fazekas II

3. Severe - extensive confluent WMLs: Fazekas III

To create a binary categorical variable from this system, if the report is described as ‘unsure’, ‘normal’ or ‘mild’ this is categorized as normal as this never requires treatment for cardiovascular risk factors. However, if there is a description of moderate or severe WMLs, the report is categorized as abnormal as these cases sometimes require treatment for cardiovascular risk factors.

Included as normal are descriptions of scattered non-specific ‘white matter dots’ or ‘foci of signal abnormality’ (unless a more defuse or specific pathology is implied) and small vessel disease described as ‘minor’, ‘minimal’ or ‘modest’.

Conversely, those cases which are described as ‘mild to moderate’, ‘confluent’, or ‘beginning to confluence’ small vessel disease are treated as abnormal.

Genetic small vessel disease, in particular Cerebral Autosomal Dominant Arteriopathy with Subcortical Infarcts and Leukoencephalopathy (CADASIL), is considered abnormal.

Mass

All the following intracranial masses are categorized as abnormal:

− Neoplasms (tumors)

Supplemental materials undergo peer review but are not copyedited, and may include typographical errors.

- Intra-axial including all primary and secondary neoplasms
- Extra-axial including all primary and secondary neoplasms
    - Pituitary adenomas included
- Lipomas included

− Tumor debulking or partial resection as this implies residual tumor (note: these are labelled as both 'encephalomalacia' and 'mass' abnormalities)

− Ependymal, subependymal or local meningeal enhancement (non-surgical) in the context of a history of an aggressive infiltrative tumor

− Abscess

− Cysts

- Retrocerebellar cyst (mega cisterna magna not included)
- Arachnoid cysts
- Pineal cysts and choroid fissure cysts
- Rathke cleft cysts

– Focal cortical dysplasia, nodular grey matter heterotopia, subependymal nodules and subcortical tubers

– Chronic subdural hematoma or hygroma (i.e., cerebrospinal fluid (CSF) equivalent)

– Perivascular spaces normal unless giant

– MRI examinations for stereotactic surgical planning alone may have very brief reports. In these scenarios it is typically evident from the clinical information provided that there is a mass e.g., surgical planning for glioblastoma.

Note that findings that typically may have minimal clinical relevance when confirmed by a neuroradiology expert, are included in this category e.g., arachnoid cyst. The rationale is that such a finding might generate a referral to a multidisciplinary team meeting for clarification clinical relevance. We consider that a referral to a multidisciplinary team meeting is a clinical intervention, and we aim to ensure that any findings that generate a downstream clinical intervention are included.

Vascular

All the following are categorized as abnormal for vascular:

– Aneurysm

Supplemental materials undergo peer review but are not copyedited, and may include typographical errors.

• including coiled aneurysms regardless of whether there is a residual neck or not

– Arteriovenous malformation

– Arteriovenous dural fistula

– Cavernoma

– Capillary telangiectasia

– Chronic / non-specific microhemorrhages

– Petechial hemorrhage

– Developmental venous anomaly

– Venous sinus thrombosis

– Vasculitis if associated with vessel changes such as luminal stenosis or vessel wall enhancement

– Arterial occlusion / flow void abnormality or absence

– Venous sinus tumor invasion (this is labelled as both 'vascular' and 'mass' abnormalities)

– Arterial stenosis. If constitutional / normal variant not included.

– Vascular-like findings which are considered normal include descriptions of sluggish flow, flow-related signal abnormalities (unless they raise the suspicion of thrombus) and vascular fenestrations.

Note that findings that typically may have minimal clinical relevance when confirmed by a neuroradiology expert, are included in this category e.g., developmental venous anomaly. The rationale is that such a finding might generate a referral to a multidisciplinary team meeting for clarification of clinical relevance. We consider that a referral to a multidisciplinary team meeting is a clinical intervention and we aim to ensure that any findings that generate a downstream clinical intervention are included.

Encephalomalacia

All the following are categorized as abnormal for encephalomalacia:

– Gliosis

– Encephalomalacia

– Cavity

Supplemental materials undergo peer review but are not copyedited, and may include typographical errors.

– Post-operative tissue changes / appearances are included as encephalomalacia

− Tumor debulking or partial resection as this implies residual tumor (note: these are labelled as both 'encephalomalacia' and 'mass'' abnormalities)

– Chronic infarct / sequelae of infarct

– Chronic hemorrhage / sequelae of hemorrhage (with / without hemosiderin staining)

– Cortical laminar necrosis

Encephalomalacia-like findings which are considered normal unless there is a clear description of related parenchymal injury include craniotomy, burr-holes, posterior fossa decompression, and 3rd ventriculostomy

Acute stroke

All the following are categorized as abnormal for acute stroke:

– Acute / subacute infarct (if demonstrating restricted diffusion)

  • Include if there are other descriptors indicating a subacute nature such as swelling even though restricted diffusion has normalized

– If a single ischemic event with both diffusion restricting and non-restricting elements then this is labelled as an 'acute stroke' abnormality (rather than an 'encephalomalacia' abnormality)

– Parenchymal post-operative restricted diffusion secondary to retraction injury

– Mitochondrial Encephalopathy with Lactic Acidosis and Stroke-like episodes (MELAS) if associated with restricted diffusion

– Hypoxic ischemic injury if associated with restricted diffusion

– Vasculitis if associated with acute / subacute infarct

– 'Mature', 'established', 'chronic' or 'old' infarcts without other descriptors are labelled as 'encephalomalacia' abnormalities

White matter inflammation

All the following are categorized as abnormal for white matter inflammation:

– Multiple sclerosis (MS) including when some plaques show cavitation (low T_1 signal)

Supplemental materials undergo peer review but are not copyedited, and may include typographical errors.

– Other demyelinating lesions including Acute Disseminated Encephalomyelitis (ADEM) and Neuromyelitis Optica spectrum disorder (NMO)

– Inflammatory lesions in Radiologically Isolated Syndrome / Clinically Isolated Syndrome

– Focal cortical thinning i.e., secondary to chronic subcortical / cortical lesions, are labelled as 'encephalomalacia' abnormalities

– Progressive Multifocal Leukoencephalopathy (PML)/ Immune Reconstitution Inflammatory Syndrome (IRIS)

– Leukoencephalopathies - congenital or acquired (including toxic)

– Encephalitis / encephalopathy if it involves the white matter, e.g. related to human immunodeficiency virus (HIV) and congenital cytomegalovirus (CMV)

– Posterior Reversible Encephalopathy Syndrome (PRES)

– Osmotic demyelination (central pontine myelinolysis/ extrapontine myelinolysis)

– Susac syndrome

– Radiation if describing white matter abnormality

– White matter changes in the context of vasculitis if clearly attributed to vasculitis.

– Amyloid-related inflammatory change / inflammatory

Atrophy

Volume loss in excess of age

General abnormality category:

In addition to these 7 specialized categories, there is a generalized 'abnormal' category. This includes reports describing any abnormality from the 7 granular categories, as well as any of the following:

<u>Hydrocephalus:</u>

– Acute

– Trapped ventricle

– Chronic / stable / improving hydrocephalus (it does not matter whether its compensated or not)

– Ventricular enlargement

Supplemental materials undergo peer review but are not copyedited, and may include typographical errors.

– normal pressure hydrocephalus (NPH)

Hemorrhage:

– Any acute / subacute hemorrhage parenchymal, subarachnoid, subdural, extradural

– Acute microhemorrhages / petechial hemorrhages

Foreign body:

– Shunts

– Clips

– Coils

– If significant metalwork is involved in skull repair e.g. in a cranioplasty (or the occasional craniotomy causing extreme intracranial MRI signal distortion)

– If craniotomies are not causing anything other than slight artefact, then these are considered normal

Extracranial:

– Total mastoid opacification / middle ear effusions

– Complete opacification / obstruction of the paranasal sinuses

– Mucosal thickening is not included

– If there is clearly a well-defined unambiguous polyp then label as abnormal.

– If ‘retention cysts’ or ‘polypoid mucosal thickening’ then label as normal. If it is something indistinguishable which could be a retention cyst / polyp then label as normal.

– Anything leading to sinus obstruction always label as abnormal.

– Calvarial / extra-calvarial masses

– Osteo-dural defects

– Encephaloceles

– Pseudomeningoceles

– Extracranial vascular abnormalities i.e., below the petrous segment e.g. cervical internal carotid artery (ICA) dissection

– Extracranial masses including lipoma or sebaceous cyst

Supplemental materials undergo peer review but are not copyedited, and may include typographical errors.

– Orbital abnormalities

- Including optic nerve pathology affecting the orbital segment of the nerve i.e., meningioma

– Cases with isolated tortuous optic nerve sheath complexes with no other features suggestive of raised intracranial pressure, are labelled as normal

– Eye prostheses and proptosis

– Pseudophakia is labelled as normal

– Bone abnormality e.g., low bone signal secondary to hemoglobinopathy

– Basilar invagination

– Hyperostosis is considered normal

– Thornwald cysts are considered normal

Intracranial miscellaneous:

– Cerebellar ectopia

– Brain herniation (e.g., through a craniectomy defect)

– Clear evidence of intracranial hypertension (e.g., prominent optic nerve sheaths AND intrasellar subarachnoid herniation)

- Isolated intrasellar subarachnoid herniation / empty sella is labelled normal
- Isolated tapering of dural venous sinuses is labelled normal

– Clear evidence of intracranial hypotension (e.g., pituitary enlargement AND pachymeningeal thickening)

- If subdural collections present, these are also labelled as ‘mass’

– Cerebral oedema or reduced CSF spaces from parenchymal swelling

– Absent or hypoplastic structures such as agenesis of the corpus callosum

– Meningeal thickening or enhancement for example in the context of neurosarcoid or vasculitis

– Enhancing or thickened cranial nerves

– Infective processes primarily involving the meninges or ependyma (i.e. ventriculitis or meningitis)

– Encephalitis if primarily involving the cortex (herpes simplex virus (HSV)/ autoimmune encephalitis)

– Excessive or unexpected basal ganglia or parenchymal calcification

– Optic neuritis involving the intracranial segments of the optic nerves or chiasmitis

– Adhesions / webs

– Pneumocephalus

– Colpocephaly

– Superficial siderosis

– Ulegyria

– Focal areas of signal intensity (FASIs) / Unidentified bright objects (UBO)

– Basal ganglia / thalamic changes in the context of metabolic abnormalities

– Neurovascular conflict fulfilling conditions of nerve distortion AND nerve root entry zone involvement

– Band heterotopia and polymicrogyria

– Hypophysitis

– Seizure related changes

– Amyotrophic lateral sclerosis (ALS).

References

38. Fazekas, F., Chawluk, J. B., Alavi, A., Hurtig, H. I., & Zimmerman, R. A. (1987). MR signal abnormalities at 1.5 T in Alzheimer's dementia and normal aging. *American journal of roentgenology*, *149*(2), 351-356.

Appendix S4

NeuroBERT versus BERT vocabulary

Supplemental materials undergo peer review but are not copyedited, and may include typographical errors.

Word	BERT	NeuroBERT
cerebellar	['ce', 're', 'bell', 'ar']	['cerebellar']
intracranial	['int', 'rac', 'ran', 'ial']	['intracranial']
hemorrhage	['ha', 'em', 'or', 'rh', 'age']	['hemorrhage']
aneurysm	['ane', 'ur', 'ys', 'm']	['aneurysm']
pons	['p', 'ons']	['pons']
suprasellar	['sup', 'rase', 'll', 'ar']	['suprasellar']
pituitary	['pit', 'uit', 'ary']	['pituitary']
infarction	['inf', 'ar', 'ction']	['infarction']
hyperintensities	['hyper', 'int', 'ens', 'ities']	['hyperintensities']
parenchyma	['paren', 'chy', 'ma']	['pa', 'renchyma']
trigeminal	['tr', 'ig', 'em', 'inal']	['trigeminal']
hippocampal	['h', 'ipp', 'ocamp', 'al']	['hippocampal']
encephalomalacia	['ence', 'phal', 'omal', 'acia']	['encephalomalacia']
hematoma	['ha', 'em', 'at', 'oma']	['hematoma']
thalamus	['thal', 'amus']	['thalamus']
subarachnoid	['sub', 'ar', 'ach', 'n', 'oid']	['subarachnoid']
cerebral	['ce', 're', 'bral']	['cerebral']
tumor	['t', 'um', 'our']	['tumor']
ischemic	['is', 'cha', 'emic']	['ischemic']
meningioma	['m', 'ening', 'i', 'oma']	['meningioma']
periventricular	['per', 'iv', 'entric', 'ular']	['periventricular']
occipital	['occ', 'ip', 'ital']	['occipital']

Comparison of WordPiece tokenization by BERT and NeuroBERT for common neuroradiological terms. By using a dedicated neuroradiological vocabulary, NeuroBERT produces far fewer word breakdowns compared to BERT when applied to domain-specific jargon.

NeuroBERT Masked Language Modelling (MLM) pretraining

Supplemental materials undergo peer review but are not copyedited, and may include typographical errors.

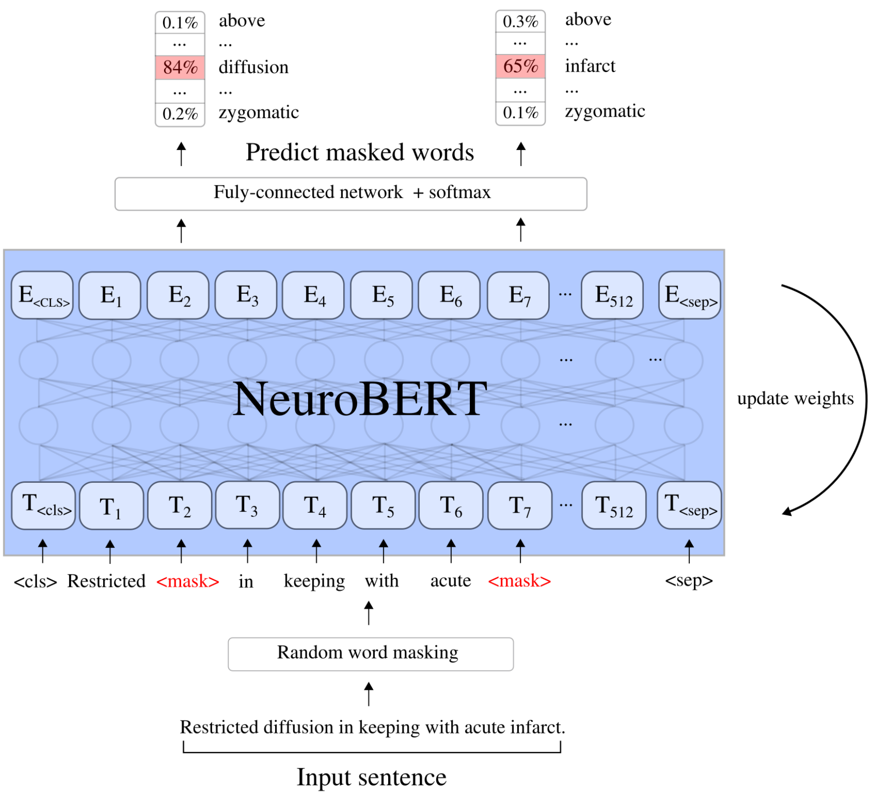

Figure S2: *Overview of our masked language modelling (MLM) training procedure for NeuroBERT. In MLM, randomly masked tokens in a sentence are predicted using surrounding context. For example, 'diffusion' and 'infarct' are masked in 'restricted diffusion in keeping with acute infarct.' The embeddings of the masked tokens are passed through a feed-forward network to predict the missing words. NeuroBERT's predictions are compared to the ground truth using cross-entropy loss, and the model weights are updated via backpropagation.*

NeuroBERT Radiology Section Matching (RSM) pretraining

Supplemental materials undergo peer review but are not copyedited, and may include typographical errors.

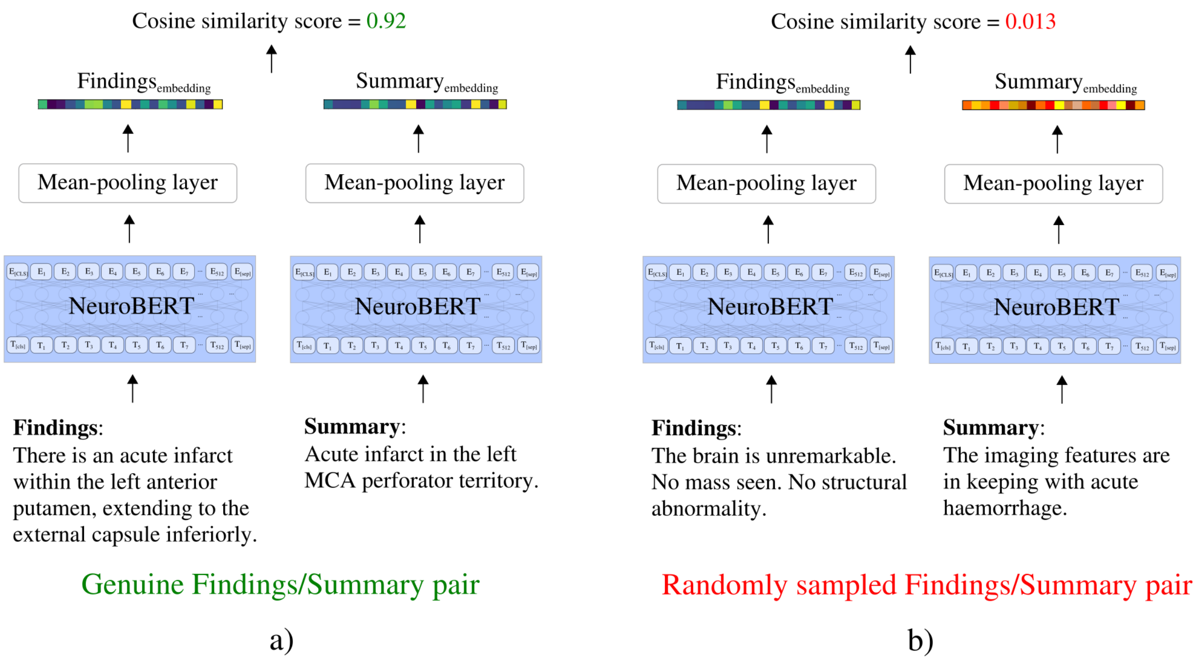

Figure S3: *Overview of our radiology section matching (RSM) procedure. RSM trains NeuroBERT to generate similar embeddings for 'Findings' and 'Summary' sections from the same report (a), and dissimilar embeddings for sections from different reports (b). Using a Siamese network with tied weights, we process section pairs, obtain embeddings via mean pooling, and compute cosine similarity. The model is trained by minimizing the MSE between the similarity score and the ground-truth label (1 for same-report pairs, 0 for random pairs).*

NeuroBERT prompt variation analysis

Supplemental materials undergo peer review but are not copyedited, and may include typographical errors.

To assess the robustness of NeuroBERT to prompt variations, including descriptive versus diagnostic phrasing, we performed an analysis by embedding several queries related to Alzheimer's disease using NeuroBERT – namely "Marked medial temporal lobe atrophy with disproportionate hippocampal volume loss", "Prominent degeneration of the hippocampus and parahippocampal regions", "Advanced medial temporal structural changes with selective hippocampal involvement", "Pronounced atrophy in medial temporal structures consistent with a degenerative process", and "Distinctive pattern of medial temporal lobe atrophy suggesting underlying neurodegeneration" - and compared their cosine similarity scores to each other and to representative diagnostic queries. To baseline these results, we also compute the pairwise cosine similarities for unrelated queries e.g., "normal study", "restricted diffusion in keeping with acute stroke" etc.

The resulting heatmap (Figure S4) shows an average cosine similarity between Alzheimer's disease-related query embeddings (whether descriptive or diagnostic) of 0.918, indicating that NeuroBERT has effectively learned to map these diverse phrases to similar semantic representations. In contrast, the average cosine similarity between Alzheimer's disease-related and unrelated query embeddings is considerably lower (~0.1). This finding implies that our retrieval performance is robust with respect to variations in query phrasing.

Supplemental materials undergo peer review but are not copyedited, and may include typographical errors.

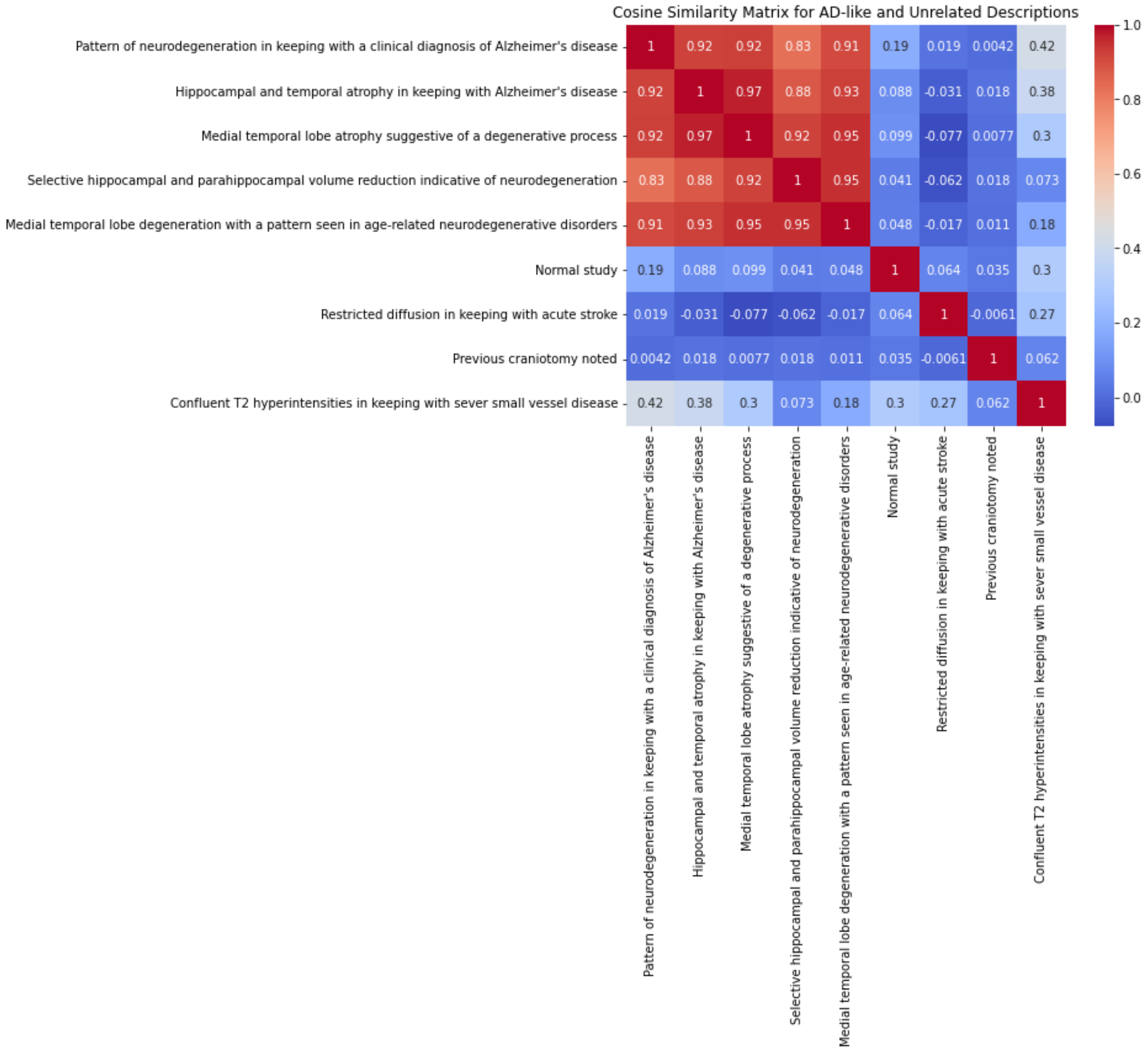

Figure S4: *Heatmap of pairwise cosine similarity scores for Alzheimer's disease–related query embeddings generated by NeuroBERT. A high average cosine similarity (0.918) is observed among queries—whether using descriptive language (e.g., "Marked medial temporal lobe atrophy with disproportionate hippocampal volume loss") or diagnostic language (e.g., "findings in keeping with Alzheimer's disease")—demonstrating the model's robustness to prompt variations. In contrast, the average cosine similarity between Alzheimer's disease-related and unrelated queries (e.g., "normal study") is much lower (~0.1), serving as a baseline.*

Supplemental materials undergo peer review but are not copyedited, and may include typographical errors.

Multi-point impression analysis

To empirically validate that our single vector embedding reliably captures multi-point impressions, we selected several representative reports from our testing set that describe multiple significant findings. For each report, we generated both relevant ("present") queries (targeting specific abnormalities mentioned in the report) and irrelevant ("absent") queries (targeting pathologies not mentioned in the report). We then embedded both the report and each query using NeuroBERT and measured the cosine similarity between each.
The following examples summarize our analysis:

Sample Report 1:
"**Findings:** There are patchy deep and periventricular cerebral white matter hyperintensities suggestive of moderate small vessel disease that exceeds age expectations. Several mature lacunar infarcts are noted in the right striatum, bilateral thalami, left putamen, and pons, without restricted diffusion. Multiple microhemorrhages are noted in the pons, thalami, and bilateral lentiform nuclei, which could be related to hypertensive angiopathy. There is no evidence of recent cortical infarct. **Impression:** Moderate to severe vascular disease with multiple lacunar infarcts and central microhemorrhages."

- Present Queries:
  - "moderate small vessel disease" → similarity: 0.7855
  - "lacunar infarcts" → similarity: 0.5320
  - "scattered microhemorrhages" → similarity: 0.4758
- Absent Queries:
  - "previous craniotomy" → similarity: -0.0249
  - "associated mass effect" → similarity: 0.0606
  - "communicating hydrocephalus" → similarity: -0.0389

Sample Report 2:
"**Findings:** There is a 45 × 44 mm parafalcine mass lesion in the left frontoparietal region extending across the midline beneath the falx and depressing the corpus callosum. It displays an outer ring of T2 intermediate signal with enhancement and central T2 hypointensity. Surrounding white matter T2 hyperintensity (vasogenic oedema) is present, with significant mass effect including a 13 mm midline shift. **Impression:** Left frontoparietal mass lesion with vasogenic oedema and significant mass effect."

- Present Queries:
  - "left frontoparietal mass lesion" → similarity: 0.7835
  - "associated mass effect" → similarity: 0.7220

Supplemental materials undergo peer review but are not copyedited, and may include typographical errors.

    - "vasogenic oedema" → similarity: 0.6491
- Absent Queries:
    - "acute infarct" → similarity: 0.0506
    - "chronic small vessel disease" → similarity: 0.0538
    - "scattered microhemorrhages" → similarity: 0.0607

These results clearly show that NeuroBERT's single vector embedding reliably captures the full spectrum of salient findings from multi-point impressions, thereby providing a robust training signal for our CNN models.

Supplemental materials undergo peer review but are not copyedited, and may include typographical errors.

Radiology section matching (RSM) task examples

Examples of true and false Findings and Summary pairs used for our self-supervised radiology section matching pre-training task.

1. **True pairs**

Findings: There is a small focus of restricted diffusion involving the posterior aspect of the left corona radiata in keeping with a subacute infarct. There are patchy areas of T_2 hyperintensity involving the subcortical/deep white matter, pons and left thalamus. There is more confluent smaller T_2 hyperintense foci within the basal ganglia and sub insular regions bilaterally representing a combination of small lacunar infarcts and prominent peri vascular spaces. Overall, the appearances are in keeping with moderate small vessel ischemic change.

Summary: Small subacute infarct involving the posterior aspect of the left corona radiata and features to suggest moderately severe small vessel ischemic change

---

Findings: There is a mass lesion of heterogenous signal within the right cerebellar hemisphere. This extends inferiorly through the foramen of Luschka with an exophytic component that compresses and displaces the medulla anteriorly and to the left. It extends superomedially to the roof and right lateral margin of the fourth ventricle. There is infiltration of the inferior right cerebellar hemisphere and the right middle cerebellar peduncles. The mass enhances heterogeneously and demonstrates peripheral partial restriction of diffusion. There is a central T_2 hyperintense non-enhancing component with free diffusion. The third and lateral ventricles are not enlarged. The supratentorial appearances are normal. There is no pathological enhancement within the spinal canal.

Summary: The imaging features are those of a high-grade right cerebellar tumor, likely ependymoma

---

Findings: There are minor generalized changes of involution with ventricular and sulcal prominence. The degree of minor and generalized volume loss is commensurate with the patient's age. There is diffuse high signal on T_2/FLAIR within the white matter of both cerebral hemispheres. this is predominantly periventricular with extension into the subcortical white matter of both frontal and parietal lobes. There is sparing of the corpus collosum and subcortical U-fibers.

Supplemental materials undergo peer review but are not copyedited, and may include typographical errors.

Summary: The imaging features are those of severe small vessel ischemic change within the white matter of both cerebral hemispheres

----

Findings: There are generalized changes of involution with ventricular and sulcal prominence. Although this volume loss is generalized there is a predominant temporal volume loss with bilateral hippocampal atrophy and reduction in volume of entorhinal cortex. The changes are asymmetrical with more marked frontal and temporal volume loss on the right. No focal abnormalities of the brain parenchyma have been identified.

Summary: The imaging features are of those of an Alzheimer pattern of volume loss predominantly affecting the temporal lobes. These changes are asymmetrical and more marked on the right

2. **False pairs**

Findings: There is a large, heterogenous lesion in the right frontal lobe with necrotic components and extensive surrounding vasogenic oedema with mass effect, sulcal effacement and partial effacement of right lateral ventricle. There is approximately 11 mm of contralateral midline shift to the left. A smaller lesion is seen at the grey-white matter junction in the left precentral gyrus, and this also has significant surrounding vasogenic oedema. These lesions are associated with abnormal susceptibility artefact in keeping with blood degradation products and they demonstrate heterogenous, mainly peripheral, enhancement. Smaller enhancing lesions are seen in the cerebellar hemispheres bilaterally. The post-contrast sequences also demonstrate small enhancing metastases in the left frontal, left medial temporal and in the left occipital lobes. Prominent linear subarachnoid enhancement is presumed all vascular. None of the lesions demonstrate any definite restricted diffusion. No bony destructive lesion is identified.

Summary: Normal intracranial appearances.

Findings: No acute intracranial abnormality is demonstrated. Specifically, there is no acute infarct. No microhemorrhages have occurred. The major intracranial vessels return their normal flow related signal voids. The intracranial appearances are unremarkable.

Summary: There is a large right intra axial heterogenous enhancing mass lesion consistent with a high-grade glioma. There is associated midline shift with dilatation of the left lateral ventricle.

Supplemental materials undergo peer review but are not copyedited, and may include typographical errors.

Findings: There are multiple partially confluent foci, hyperintense on the T_2 sequences, with no restriction on the DWI, located in the deep and subcortical frontoparietal white matter of both frontal cerebral hemispheres, with no evidence of involvement of the U-fibers. The cerebellum, brainstem and corpus callosum are unremarkable. The described findings are more likely to be of vascular or residual origin than of demyelinating (multiple sclerosis type) origin. The ventricles and cortical sulci are of normal size and appearance. Normal cranio-cervical junction. There is no fluid in the mastoid air cells

Summary: Solitary 5mm cystic lesion in the left para-hippocampal gyrus. This probably represents a small neuroglial cyst.

Findings: The cranio-cervical junction is normal. The brainstem and cerebellum are unremarkable. The cerebral hemispheres are normal in morphology and signal intensity pattern. There is no evidence of intracranial ischemic or hemorrhagic lesions. There is no evidence of intracranial solid or cystic lesions. The ventricular system is normal in size.

Summary: The appearances are those of longstanding compensated communicating hydrocephalus.

Supplemental materials undergo peer review but are not copyedited, and may include typographical errors.

Appendix S5

Metric definitions

Cosine similarity.

Given an image embedding $\mathbf{x}\in\mathbb{R}^d$ and a text embedding $\mathbf{y}\in\mathbb{R}^d$, their cosine similarity is

$$\mathrm{sim}(\mathbf{x},\mathbf{y}) = \frac{\mathbf{x}^{\mathrm{T}}\mathbf{y}}{\|\mathbf{x}\|_2\|\mathbf{y}\|_2} \in [-1,1]$$

We then clamp negative values to zero so that

$$\mathrm{score}(\mathbf{x},\mathbf{y}) = \max\big(0, sim(\mathbf{x},\mathbf{y})\big) \in [0,1]$$

which (a) removes any notion of "anti-alignment" by treating all negative similarities as equally irrelevant, (b) preserves the ranking induced by raw cosine, and (c) yields scores interpretable as "pseudo-probabilities."

Zero-shot classification.

For each exam we compute

$$s = \mathrm{score}(\mathbf{x},\mathbf{y}_{\mathrm{query}})$$

where $\mathbf{x}$ is the image embedding and $\mathbf{y}_{\mathrm{query}}$ is the embedding of a class-defining sentence (e.g. "this is a normal study"). These scores in [0,1] are then used to build ROC curves or thresholds for binary decisions.

Visual-semantic retrieval.

For a text query t_{query} we score every database image x_i by

$$s_i = \mathrm{score}\big(\mathbf{x}_{i,}\mathbf{y}_{\mathrm{query}}\big),$$

sort in descending order of s_i, and return the top K.

Precision@K.

Let $R \subseteq \{1,\ldots,N\}$ be the indices of truly relevant images. Denote the top K retrieved indices by $\{r_1,\ldots,r_k\}$. Define the indicator

$$I(i) = \begin{cases} 1, & r_i \in R, \\ 0, & \text{otherwise} \end{cases}$$

Supplemental materials undergo peer review but are not copyedited, and may include typographical errors.

Then

$$\text{Precision@}K = \frac{1}{K}\sum_{i=1}^{K} I(i)$$

i.e. the fraction of relevant cases among the first *K* results.

Why These Metrics Are Appropriate

- **Scale invariance & semantic alignment.** Cosine similarity measures only the angle between embeddings, making it robust to differences in vector norms that arise from separate training dynamics in our image and text encoders. In a joint embedding space trained to align modalities, orientation directly reflects conceptual compatibility.
- **Clipping to [0,1].** Treating all negative (“anti-aligned”) pairs as equally irrelevant is both intuitive—no reason to distinguish degrees of negative affinity—and standard practice in leading frameworks (e.g. Radford et al. 2021, CLIP).
- **Unified scoring for classification & retrieval.** The same score function underlies both tasks, avoiding any metric mismatch.
- **Clinical relevance of Precision@K.** Radiologists can only review a handful of prior cases under time pressure; Precision@15 directly captures system utility in real-world workflows. Note that using a smaller K (e.g., 1 or 5) would artificially inflate precision by focusing solely on the highest-confidence retrievals.

Supplemental materials undergo peer review but are not copyedited, and may include typographical errors.

Appendix S6

NeuroBERT training

All language modeling tasks were performed using Hugging Face version 4.12.5 with two NVIDIA RTX 2080 GPUs. The WordPiece tokenizer, MLM, and RSM tasks were trained using all reports in the unlabeled training set (N = 50,523). The tokenizer vocabulary size was set to 10,000, with a minimum token frequency of 10. For MLM, the sequence length was 128, with 15% of tokens randomly masking. The model was trained for 250 epochs with mini-batches of 64 sequences, with early stopping based on loss on the validation set (N = 6,315). The model configuration with the lowest validation loss served as the initial configuration for RSM training.

For RSM, regular expressions were used to separate *Findings* and *Summary* sections, and true and false pairs were randomly sampled (examples in Appendix S4). The minibatch size was 16, and the learning rate was 10^{-6} with a linear warm-up of 100 steps. The training objective was MSE between cosine similarity scores and ground-truth labels. The model configuration with the lowest validation loss was adopted as the final NeuroBERT.

CNN training

CNN models were adapted from the DenseNet121 implementation in Project MONAI. Training was performed using PyTorch 1.7.1 with two NVIDIA GPUs. The Adam optimizer (39) was used with an initial learning rate of 10^{-4}, reduced by a factor of 10 after every 5 epochs without improvement in validation loss. Early stopping (patience = 5) was applied such that each sequence-specific model converged based on validation performance.

Note that this two-step training procedure can be considered cross-modal distillation, in which a pretrained model in one modality (NeuroBERT) served as the teacher, and its frozen embeddings supervised a student network in another modality (the sequence-specific

Supplemental materials undergo peer review but are not copyedited, and may include typographical errors.

CNNs). Importantly, neither teacher nor student had access to the held-out test set, ensuring no risk of data leakage or bias in assessing generalization.

We measured the training (plus validation) time for each of the eight MRI sequence models as follows:

- Ax_T1_gad: 26m 30s (training) + 2m 14s (validation) per epoch over 32 epochs ≈ 15.3 hours
- Ax_FLAIR: 22m 50s + 1m 56s per epoch over 30 epochs ≈ 12.4 hours
- Ax_T2: 2h 06m 29s + 9m 37s per epoch over 26 epochs ≈ 59.0 hours
- Cor_T1: 28m 19s + 2m 05s per epoch over 31 epochs ≈ 15.7 hours
- Cor_T1_Gad: 18m 31s + 1m 36s per epoch over 30 epochs ≈ 10.1 hours
- Cor_FLAIR: 1h 08m 19s + 5m 16s per epoch over 31 epochs ≈ 38.0 hours
- GRE: 53m 06s + 4m 28s per epoch over 32 epochs ≈ 30.7 hours
- DWI: 1h 38m 57s + 7m 26s per epoch over 32 epochs ≈ 56.7 hours

The combined training and validation time across all sequence models is approximately 238 hours.

Inference Analysis:

For inference, we measured the end-to-end processing time per image—including pre-processing, CNN-based embedding generation, and cosine similarity computation with precomputed text embeddings—using our NVIDIA GPU. On average, the inference time per image is 0.161 seconds.

Using NVIDIA's NVML (NVIDIA Management Library) via the Python package pynvml, we recorded the GPU power draw during inference and estimated an average energy consumption of approximately 7.77 Joules per query. This corresponds to an average power draw of roughly 48 Watts during inference.

Model explainability

Supplemental materials undergo peer review but are not copyedited, and may include typographical errors.

A method was implemented to scrutinize our framework's predictions using guided backpropagation (40). The derivative of the similarity score for a query sentence was computed and backpropagated through the image encoder, retaining only positive error signals. This process yielded a gradient array highlighting regions that would significantly alter the text-image similarity score if changed slightly.

Guided backpropagation generated heatmaps matching the input image dimensions. To aid radiologists, slice-wise saliency lineouts were generated by computing the maximum gradient per slice, and the heatmap for the most influential slice (the slice with the highest gradient) was displayed. To suppress noise-induced high gradients, the procedure was repeated 50 times with Gaussian noise added (mean = 0, standard deviation = .1), and the mean aggregated gradient array was used (41).

References:

39. Kingma, D. P. (2014). Adam: A method for stochastic optimization. *arXiv preprint arXiv:1412.6980*.

40. Springenberg, J. T., Dosovitskiy, A., Brox, T., & Riedmiller, M. (2014). Striving for simplicity: The all convolutional net. *arXiv preprint arXiv:1412.6806*.

41. Smilkov, D., Thorat, N., Kim, B., Viégas, F., & Wattenberg, M. (2017). Smoothgrad: removing noise by adding noise. *arXiv preprint arXiv:1706.03825*.

Supplemental materials undergo peer review but are not copyedited, and may include typographical errors.

Appendix S7

Text-vision subgroup classification

Supplemental materials undergo peer review but are not copyedited, and may include typographical errors.

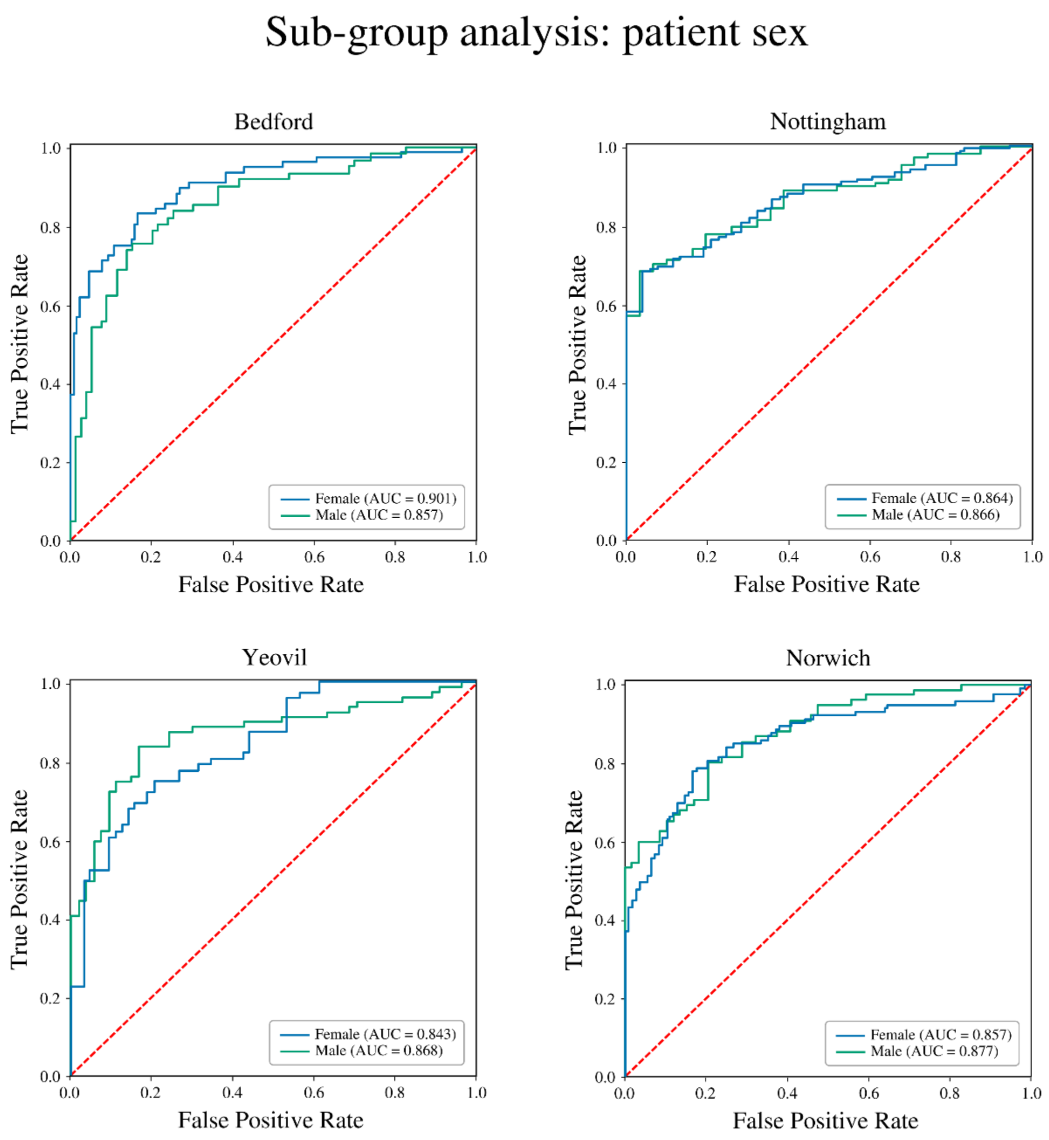

Figure S5: *Sub-group analysis by patient sex. ROC curves for the four external hospitals are shown, comparing classification performance between male and female cohorts. At Bedford, the AUCs were 0.90 (95% CI: [0.86, 0.94] for Female and 0.86 [0.80, 0.91] for Male; at Nottingham, the AUCs were 0.86 [0.82, 0.91] for Female and 0.87 [0.81, 0.92] for Male; at Yeovil, the AUCs were 0.84 [0.78, 0.90] for Female and 0.87 [0.81, 0.92] for Male; and at Norwich, the AUCs were 0.86 [0.81, 0.90] for Female and 0.88 [0.82, 0.93] for Male.*

General

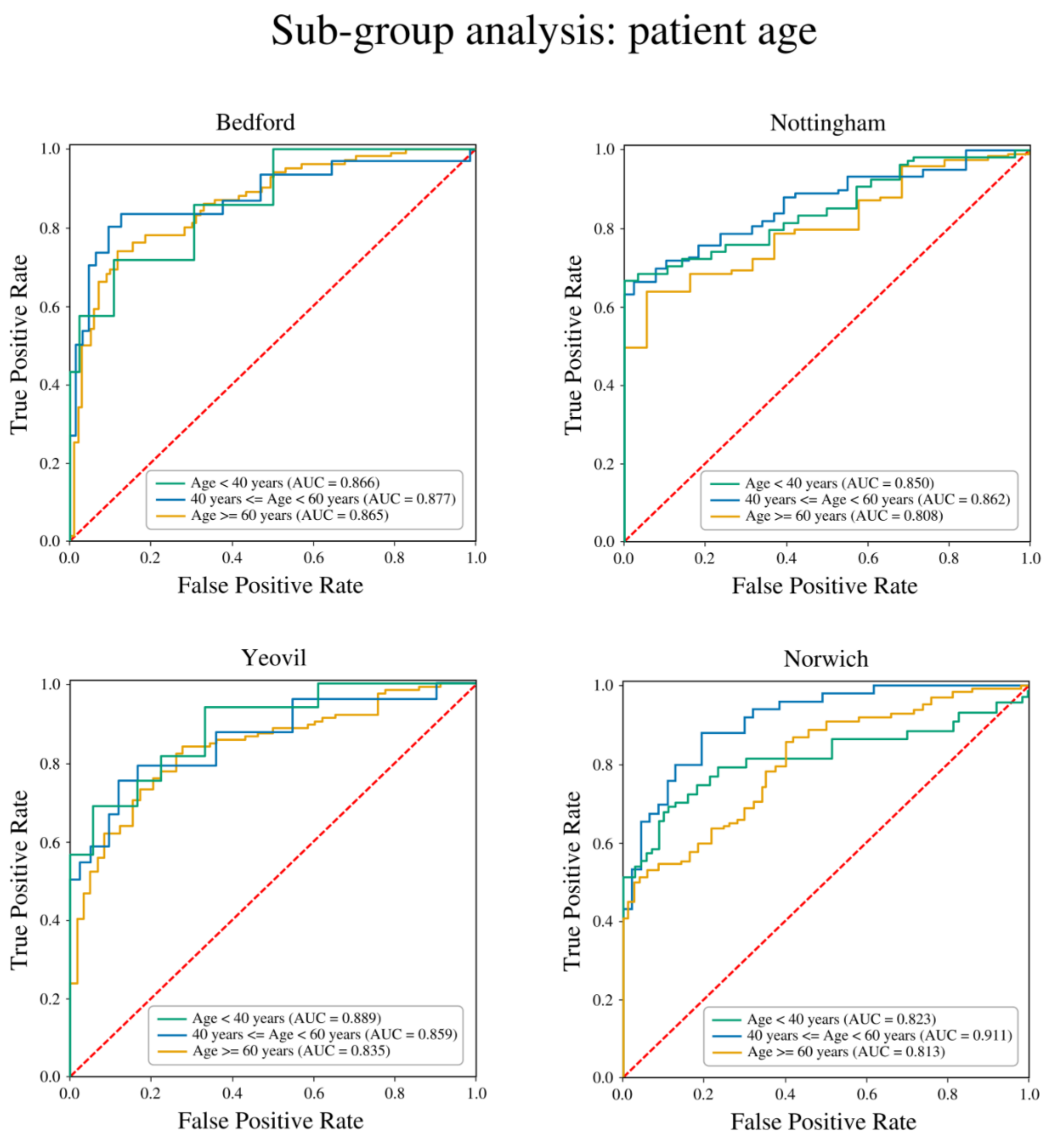

Figure S6: *Sub-group analysis by patient age. ROC curves for the four external hospitals are shown for three age brackets (<40, 40–60, and >60 years). Within each hospital the respective AUCs were 0.87; 95% CI: [0.78, 0.96], 0.88 [0.81, 0.94], and 0.87 [0.82, 0.91] in Bedford; 0.850 [0.77, 0.93], 0.86 [0.80, 0.92], and 0.81 [0.74, 0.87] in Nottingham; 0.89 [0.80, 0.98], 0.86 [0.78, 0.94], and 0.84 [0.78, 0.89] in Yeovil; and 0.82 [0.75, 0.89], 0.91 [0.86, 0.97], and 0.81 [0.75, 0.88] in Norwich.*

Supplemental materials undergo peer review but are not copyedited, and may include typographical errors.

Sub-group analysis: MRI scanner manufacturer

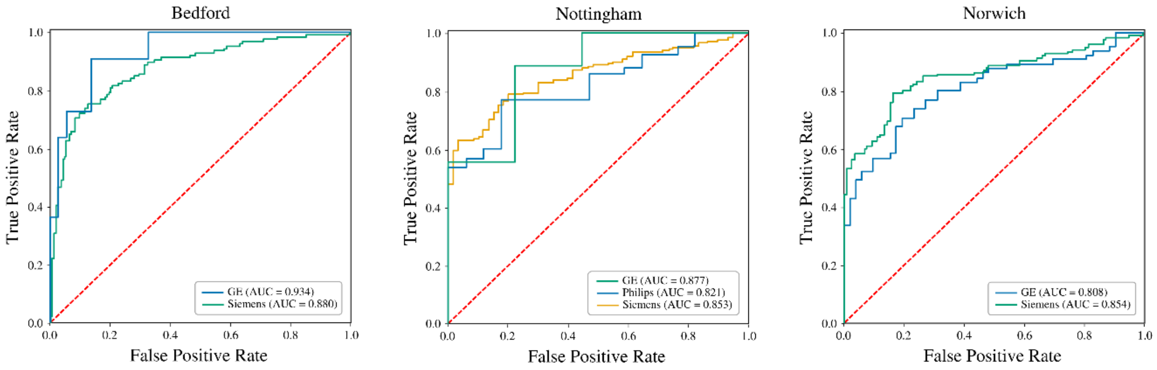

Figure S7: *Sub-group analysis by MRI scanner manufacturer. For external sites with multiple manufacturers represented (Bedford, Nottingham, and Norwich), ROC curves compare performance across GE, Philips, and Siemens scanners. Bedford showed AUCs of 0.93 [0.86, 1.00] for GE and 0.88 [0.84, 0.92] for Siemens; Nottingham showed AUCs of 0.88 (95% CI: [0.73, 1.00]) for GE, 0.82 [0.74, 0.90] for Philips, and 0.85 [0.81–0.90] for Siemens; and Norwich showed AUCs of 0.81 [0.74, 0.88] for GE and 0.85 [0.81, 0.90] for Siemens.*

Supplemental materials undergo peer review but are not copyedited, and may include typographical errors.

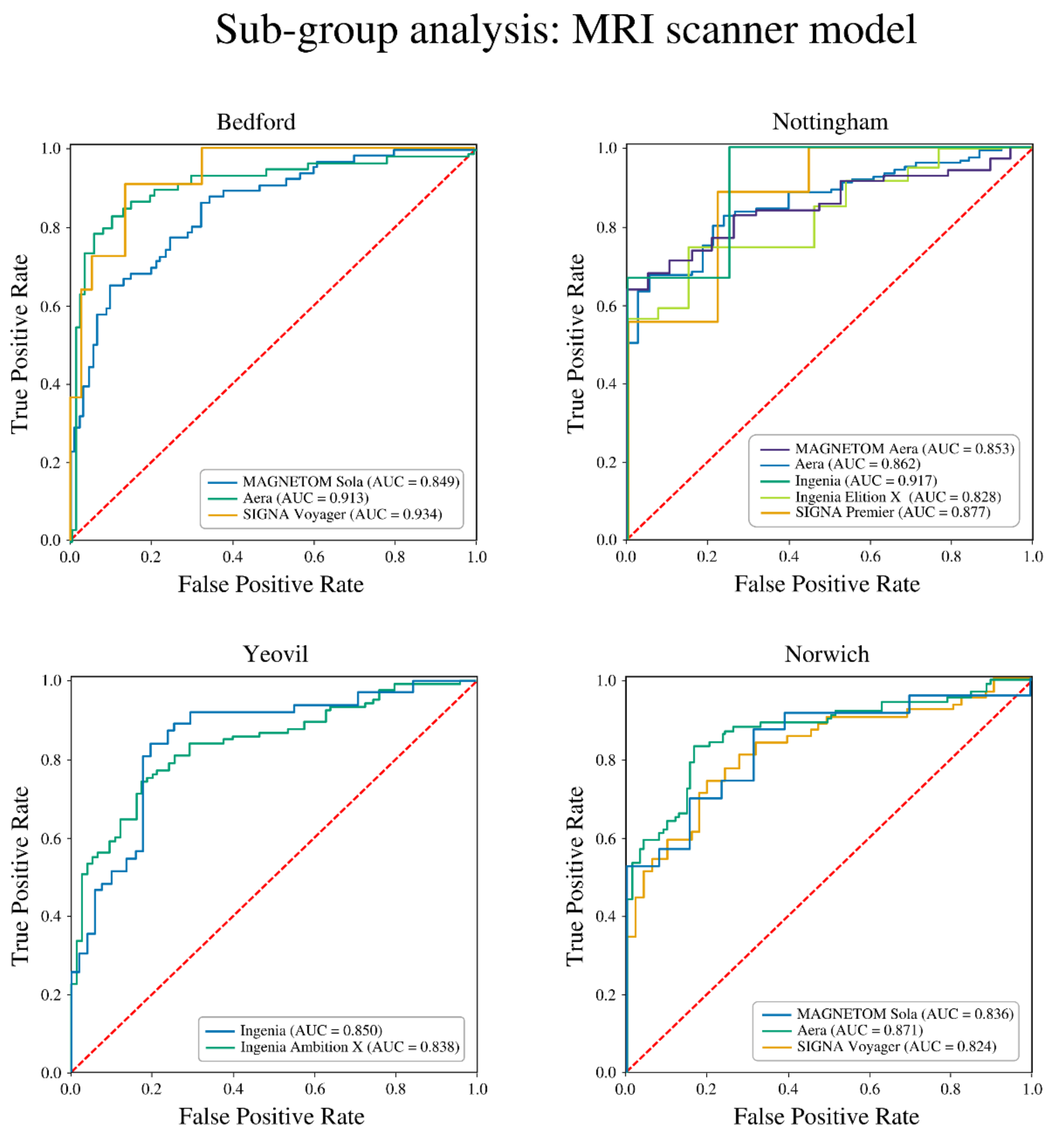

Figure S8: *Sub-group analysis by MRI scanner model. ROC curves for Bedford (top-left), Nottingham (top-right), Yeovil (bottom-left), and Norwich (bottom-right) compare performance across different scanner models. Bedford showed AUCs of 0.85 (95% CI: [0.79, 0.91]) for MAGNETOM Sola, 0.91 [0.87, 0.96] for Aera, and 0.93 [0.86, 1.00] for SIGNA Voyager; Nottingham showed AUCs of 0.85 [0.78, 0.93] for MAGNETOM Aera, 0.86 [0.81, 0.92] for Aera, 0.92 [0.75, 1.00] for Ingenia, 0.83 [0.74, 0.91] for Ingenia Elition X, and 0.88 [0.73, 1.00] for SIGNA Premier; Yeovil showed AUCs of 0.84 [0.78, 0.89] for Ingenia Ambition X and 0.85 [0.78, 0.92] for Ingenia; and Norwich showed AUCs of 0.84 [0.72, 0.96] for MAGNETOM Sola, 0.87 [0.83, 0.92] for Aera, and 0.82 [0.76, 0.89] for SIGNA Voyager.*

Supplemental materials undergo peer review but are not copyedited, and may include typographical errors.

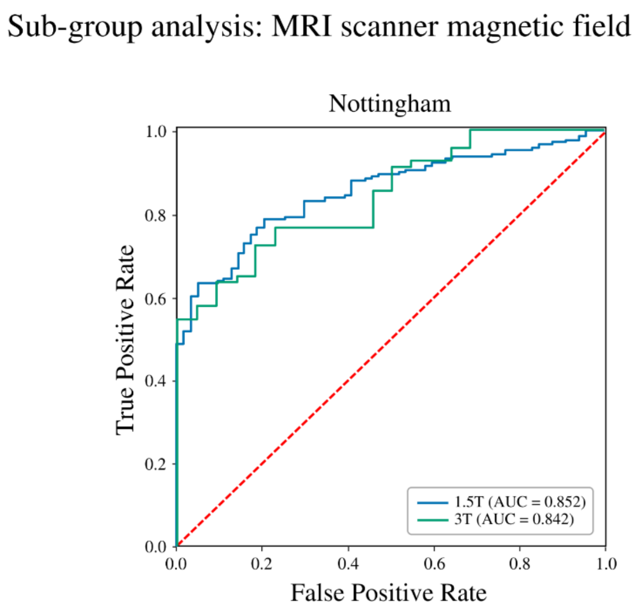

Figure S9: *Sub-group analysis by MRI magnetic field strength. At Nottingham, where both 1.5T and 3T data were available, ROC curves compare performance between the two field strengths, with AUCs of 0.85 (95% CI: [0.81, 0.89]) for 1.5 T and 0.84 [0.77, 0.92] for 3 T.*

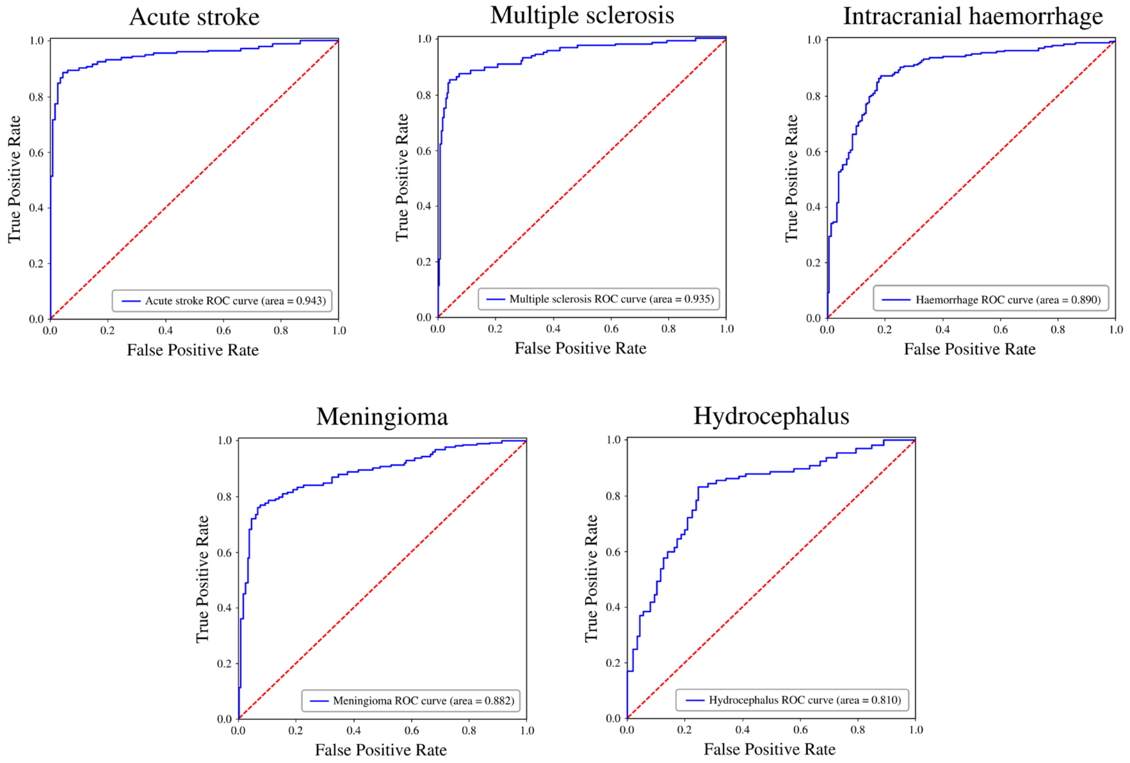

Figure S10: *ROC curve when utilizing our ensemble approach to classify brain MRI examinations as either 'acute stroke' or 'no acute stroke' (top left; AUC = 0.94; 95% CI:*

Supplemental materials undergo peer review but are not copyedited, and may include typographical errors.

[0.91, 0.98]), ‘multiple sclerosis’ or ‘no multiple sclerosis’ (top middle; AUC = 0.94 [0.90, 0.97], ‘intracranial hemorrhage’ or ‘no intracranial hemorrhage’ (top right; AUC = 0.89 [0.85, 0.93]), ‘meningioma’ or ‘no meningioma’ (bottom left; AUC = 0.88 [0.84, 0.93]), and ‘hydrocephalus’ or ‘no hydrocephalus’ (bottom right; AUC = 0.81 [0.76, 0.86]).

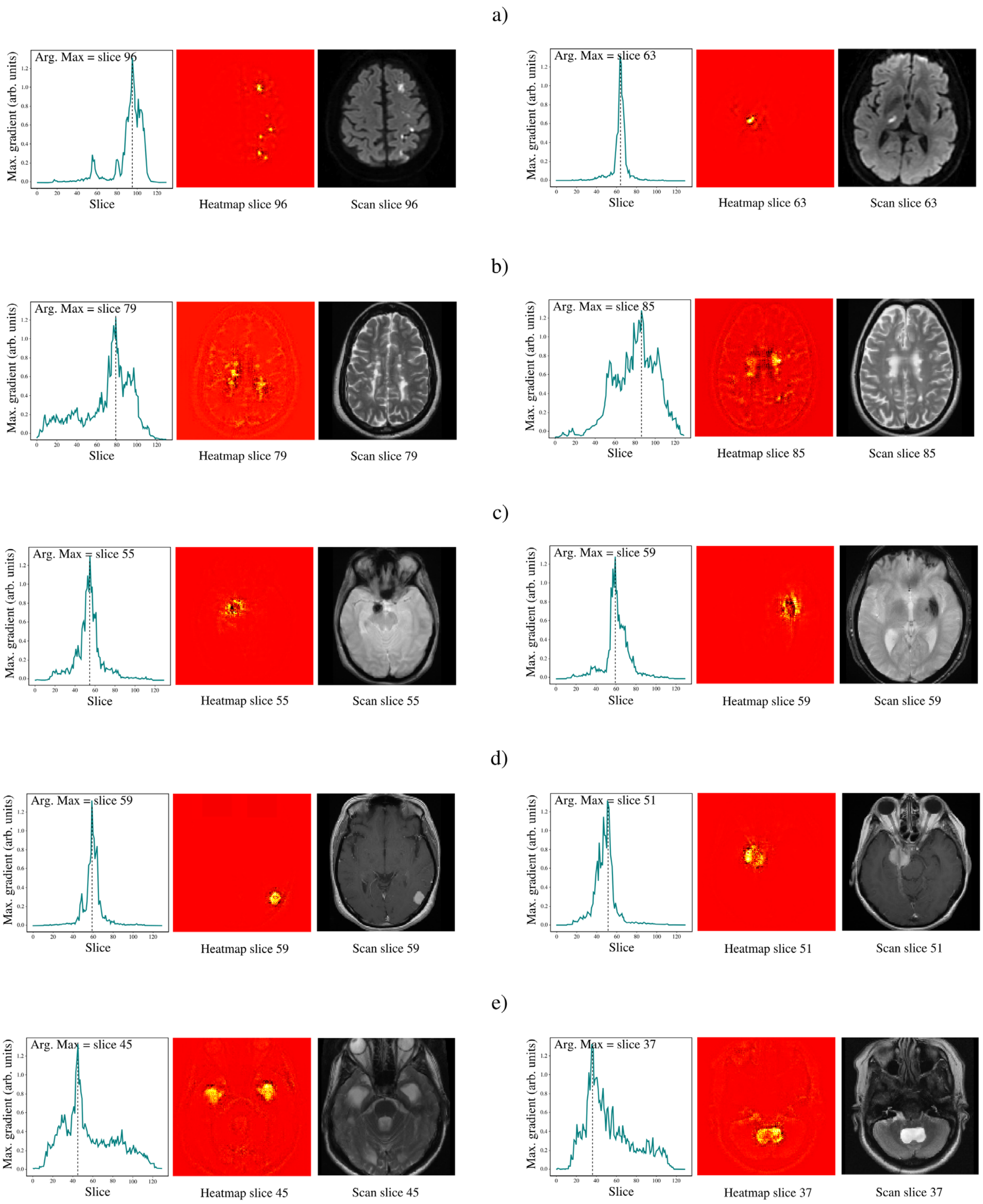

Figure S11: *Smooth guided backpropagation applied to scans from the five specialized classification test sets. a) acute stroke (acute infarction on DWI imaging); b) multiple sclerosis (periventricular Dawson’s fingers on T_2-weighted imaging); c) intracranial*

General

Supplemental materials undergo peer review but are not copyedited, and may include typographical errors.

hemorrhage (susceptibility from blood products on GRE imaging); d) meningioma (avid and homogenous extra-axial enhancement on T_1*-weighted (post-contrast) imaging); e) hydrocephalus (enlarged ventricles on* T_2*-weighted imaging).*

Supplemental materials undergo peer review but are not copyedited, and may include typographical errors.

Appendix S8

a)

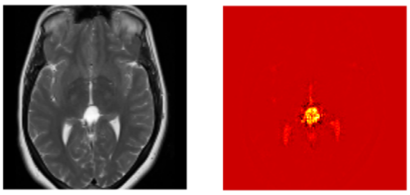

Findings:
There is a simple pineal cyst measuring 17 mm in diameter. No convex features. No solid components and no restricted diffusion. There is mild anterior displacement of the tectal plate. There is no abnormal enhancement seen.

Summary:
Simple 17 mm pineal cyst without complex features. The ventricles are non-dilated and there is no evidence of hydrocephalus.

b)

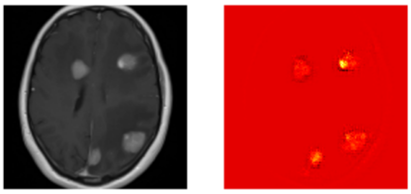

Findings:
There are numerous large enhancing masses noted. Some of the lesions are intra-axial including a 2.7 cm left frontal mass. There is a 2.8 cm right periventricular lesion centred in the head of the right caudate with some suggestion of adjacent subependymal enhancement along the margin of the right frontal horn. Several other lesions appear dural based including a left 3.2 centimetre parietal mass and left medial parieto-occipital parietal mass, abutting the postero aspect of the superior sagittal sinus, but without clearly encroaching on the flow-void.
Further smaller lesions are noted in the left temporal lobe and right frontal lobe.

Summary:
There are numerous large intra and extra-axial masses involving the cerebral and cerebellar hemispheres, which in the clinical context is likely to represent metastatic deposits.

c)

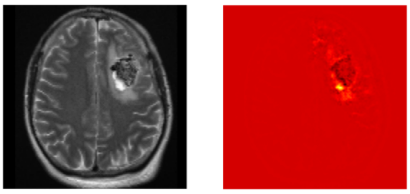

Findings:
There is a left fronto-parietal mini craniotomy. The left frontal lobe peripheral resection cavity is noted with associated T2 hyperintense/T1 isointense fluid and multiple foci of low signal consistent with air and gliadel wafers. There is T2 hyperintensity within the white matter of the left frontal lobe adjacent to the resection. There is some minimal linear areas of T1 hyperintensity at the deep and posterior margin of resection.

Summary:
The left frontal glioblastoma resection cavity is noted. Although potentially masked by minor areas of T1 hyperintensity, there is no residual gadolinium enhancement evident at this site of resection. Non specific adjacent T2 hyperintensity is noted within the white matter.

d)

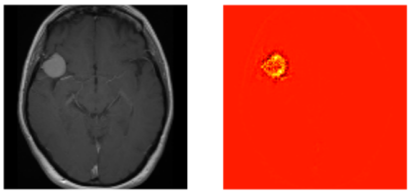

Findings:
There are stable appearances to the meningioma within the right Sylvian fissure since the previous study (maximum dimensions = 2.4 cm). There is only minor distortion of the adjacent anterior temporal lobe - brain architecture is otherwise well preserved. No further intracranial abnormality is shown.

Summary:
No interval change to the known right-sided meningioma.

e)

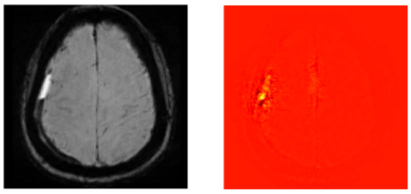

Findings:
Comparison is made with the CT head examination dated [ANONYMISED]. There remains a mixed-signal shallow right cerebral convexity subdural collection. There is smooth enhancement of the dura. No focal mass lesion, abnormal signal or focal enhancement is demonstrated to suggest an underlying cause for the recurrent haemorrhage. Slight mass effect persists (partial sulcal effacement is again noted) but there is no midline shift and the basal cisterns are patent.

Summary:
Right cerebral convexity subdural collection, as described.

f)

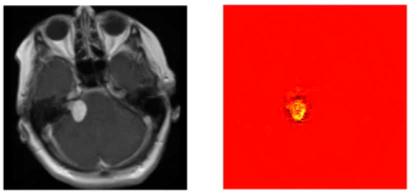

Findings:
There is a right cerebello pontine angle cistern and IAM lesion which is of intermediate T1 and T2 signal, undergoing moderate and slightly heterogeneous gadolinium enhancement. It measures 2.1cm parallel to the petrous pyramid x 1.3cm (perpendicular to the petrous pyramid). In its extra meatal portion it measures 2.2cm (cranio caudal). It does not quite reach the fundus of the right IAM. There is moulding of the right middle cerebellar peduncle. There is some patchy hemispheric white matter T2 hyperintensity. The right cerebello pontine angle cistern lesion distorts the root entry zone of the right trigeminal nerve. There is no other lesion in the line of the right trigeminal nerve.

Summary:
There is a right cerebello pontine angle cistern and IAM lesion consistent with a vestibular schwannoma. There is associated impingement on the right trigeminal nerve.

Figure S12: *Examples of correctly retrieved images using our visual-semantic database search technique. Heatmaps generated by guided backpropagation are also shown, along*

Supplemental materials undergo peer review but are not copyedited, and may include typographical errors.

with the corresponding neuroradiology report for each examination (which was not used for image retrieval). a) retrieval task: find image examples of a pineal cyst; correctly retrieved example: a 17mm pineal cyst; b) retrieval task: find image examples of metastatic deposits; correctly retrieved example: numerous cerebral metastatic deposits; c) retrieval task: find image examples of a post-surgical resection cavity; correctly retrieved example: a left frontal post-surgical resection cavity; d) retrieval task: find image examples of a meningioma; correctly retrieved example: a right sylvian fissure meningioma; e) retrieval task: find image examples of a hematoma; correctly retrieved example: a right convexity subdural hematoma; f) retrieval task: find image examples of a vestibular schwannoma; correctly retrieved example: a right cerebello-pontine vestibular schwannoma.

Supplemental materials undergo peer review but are not copyedited, and may include typographical errors.

a)

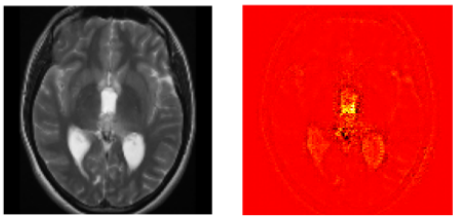

Findings:
There is an avidly enhancing well-circumscribed mass measuring 1.6 x 2.4 x 1.6 cm centred on the pineal gland containing foci of cystic change. Immediately adjacent to the lesion there is an ill-defined area of T2 hyperintensity and swelling within the posteromedial aspect of the left thalamus and hippocampal tail measuring approximately 1.8 x 1.5cm on axial images. This does not demonstrate contrast enhancement. The lack of signal change within the right thalamus makes reactive oedema a less likely explanation for this appearance

Summary:
Large pineal gland tumour with moderate dilatation of the lateral and third ventricles.

b)

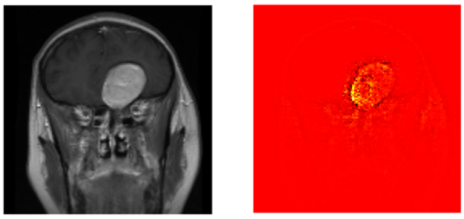

Findings:
There is a fat signal mass (5.6cm(AP) x 5.2cm(CC) x 6cm (transverse)) in the left side of the suprasellar cistern, that extends anteriorly into the subfrontal region and posterosuperiorly invaginates into the basal ganglia region of the left cerebral hemisphere. At it's inferomedial aspect, it lies medial to the anterior falx which is displaced to the right of the midline. More superomedially, the lesion itself crosses the mid line and displaces the genu of the corpus callosum posteriorly. The posterior aspect of the mass causes some distortion of the third and right lateral ventricle. The medial aspect of the lesion also impinges on and distorts the left pre-chiasmatic optic nerve and lies along the path of the left optic tract.

Summary:
Appearances are consistent with a left frontal/suprasellar dermoid as described.

c)

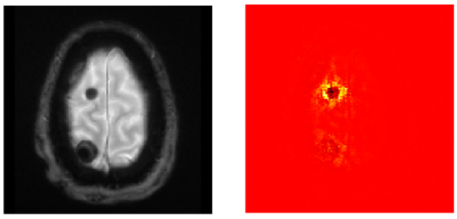

Findings:
There has been a previous extensive right sided craniotomy, beneath which there is right anterior and lateral temporal lobe damage and bilateral frontal lobe traumatic damage. There is no evidence of additional parenchymal injury or deep white matter micro-bleeds. No other potential seizure focus is identified.

Summary:
Previous frontal and temporal lobe damage, as described.

d)

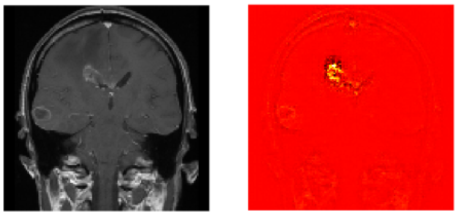

Findings:
There is a large heterogeneous 4.7x4.3x3 cm mass which is centred around the right frontal horn, with involvement of the caudate, and crossing the midline across the corpus callosum. Mass shows heterogeneous peripheral enhancement, with some ill-defined enhancement extending superiorly. Mass shows some peripheral low to intermediate T2 signal with corresponding faintly restricted diffusion suggestive of high cellularity. There is marked surrounding oedema. There is 7mm of midline shift to the left, with compression of the adjacent ventricles. There is a second 20mm peripherally enhancing lesion with mild surrounding oedema in the right temporal lobe.

Summary:
Multifocal lesions, with dominant lesion crossing the midline across corpus callosum. Differential diagnosis would include multifocal GBM or lymphoma; metastasis is less likely.

e)

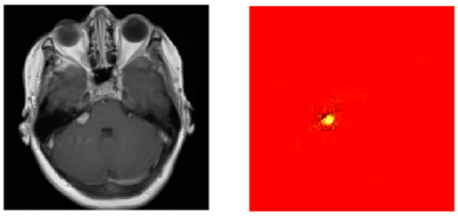

Findings:
There has been no interval change in relation to the size or appearance of the homogenously enhancing right petrous ridge mass lesion. This retains a maximum axial dimension of 12 mm. There remains minor intrameatal extension.

Summary:
Static appearance of the presumed right-sided petrous ridge meningioma.

Figure S13: *Examples where our text-vision model retrieved images which do not contain to the pathology requested; in each case, ambiguous or morphologically similar pathologies were returned. Heatmaps generated by guided backpropagation are also shown, along with the corresponding radiology report for each examination (which was not used for image retrieval). a) retrieval task: "find image examples of a pineal cyst"; incorrectly retrieved example: the image shows a pineal gland; b) retrieval task: "find image examples of*

Supplemental materials undergo peer review but are not copyedited, and may include typographical errors.

metastatic deposits"; incorrectly retrieved example: the image shows a dermoid, which has similar imaging characteristics to a meningioma; c) retrieval task: "find image examples of a hematoma"; incorrectly retrieved example: an image showing susceptibility artefacts on GRE due to bolts holding the patient's craniotomy together which has been mistaken for susceptibility resulting from a hematoma; d) retrieval task: "find image examples of metastases"; incorrectly retrieved example: the image shows a presumed glioblastoma or lymphoma (although metastasis is in the differential diagnosis); e) retrieval task: "find image examples of a vestibular schwannoma"; incorrectly retrieved example: the image shows a right-sided petrous ridge meningioma (although in this location, vestibular schwannoma is in the differential diagnosis). Note: using radiological left and right.

Supplemental materials undergo peer review but are not copyedited, and may include typographical errors.

Appendix S9

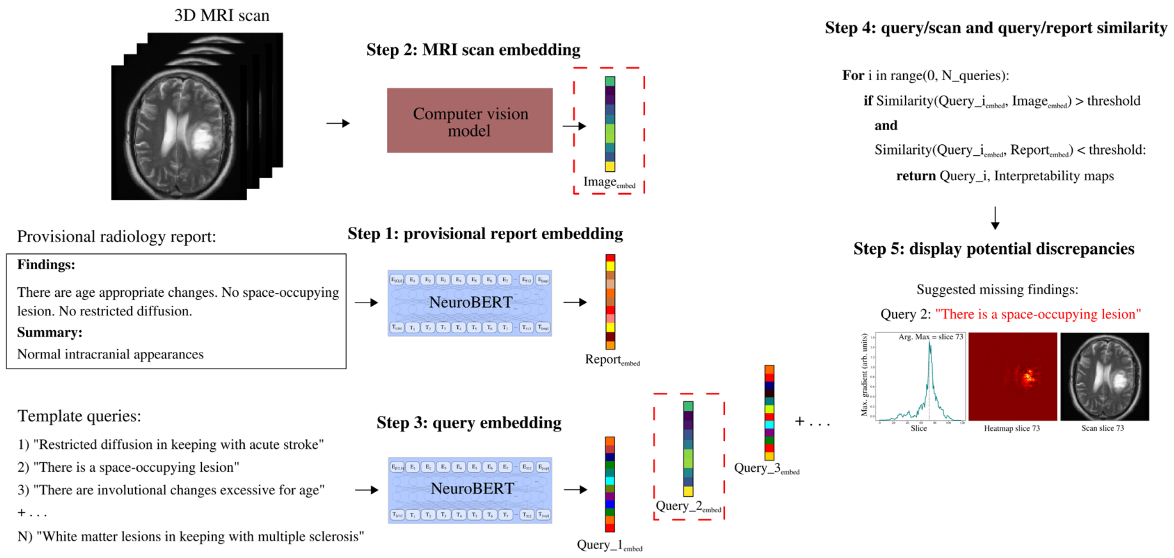

Figure S14: *Overview of our proposed approach to detect errors in provisional radiology reports. Before finalizing a radiologist's provisional report, it could undergo an additional validation step through our framework. Step 1) the report would be encoded into a 768-dimensional embedding by NeuroBERT. Step 2) the MRI scans from the examination would be similarly processed into embeddings by the corresponding single-sequence computer vision models. Step 3) A series of template queries, representing key pathological findings (such as 'acute stroke' or 'space occupying lesion'), would be encoded into the same vector space. Step 4) the framework would then calculate the cosine similarity between the query embeddings and both the scan embeddings and the provisional report embedding. Step 5) discrepancies highlighted by high similarity with the scan but low similarity with the provisional report could signal potential oversights. These flagged queries, along with their heatmaps, would be presented to radiologists for a focused review of the implicated slices.*

General
